# Microvesicle-camouflaged biomimetic nanoparticles encapsulating a metal-organic framework for targeted rheumatoid arthritis therapy

**DOI:** 10.1186/s12951-022-01447-0

**Published:** 2022-06-03

**Authors:** Yao Wang, Ming Jia, Xiu Zheng, Chenglong Wang, Yun Zhou, Hong Pan, Yan Liu, Ji Lu, Zhiqiang Mei, Chunhong Li

**Affiliations:** 1 Department of Pharmaceutical Sciences, School of Pharmacy, Southwest Medical University, 1-1 Xianglin Road, Luzhou, 646000 Sichuan People’s Republic of China; 2 School of Medical Information and Engineering, Southwest Medical University, Luzhou, Sichuan China; 3 Center for Medical Information and Modern Educational Technology, Southwest Medical University, Luzhou, Sichuan China; 4 Department of Medicinal Chemistry, School of Pharmacy, Southwest Medical University, 1-1 Xianglin Road, Luzhou, 646000 Sichuan People’s Republic of China; 5 The Research Center for Preclinical Medicine, Southwest Medical University, 1-1 Xianglin Road, Luzhou, 646000 Sichuan People’s Republic of China

**Keywords:** Rheumatoid arthritis, Metal–organic framework, Microvesicles, Folate receptor

## Abstract

**Background:**

Methotrexate (MTX) has been highlighted for Rheumatoid arthritis (RA) treatment, however, MTX does not accumulate well at inflamed sites, and long-term administration in high doses leads to severe side effects. In this study, a novel anti-RA nanoparticle complex was designed and constructed, which could improve the targeted accumulation in inflamed joints and reduce side effects.

**Results:**

Here, we prepared a pH-sensitive biomimetic drug delivery system based on macrophage-derived microvesicle (MV)-coated zeolitic imidazolate framework-8 nanoparticles that encapsulated the drug methotrexate (hereafter MV/MTX@ZIF-8). The MV/MTX@ZIF-8 nanoparticles were further modified with 1,2-distearoyl-*sn*-glycero-3-phosphoethanolamine-*N*-[folate (polyethylene glycol)-2000] (hereafter FPD/MV/MTX@ZIF-8) to exploit the high affinity of folate receptor β for folic acid on the surface of activated macrophages in RA. MTX@ZIF-8 nanoparticles showed high DLE (~ 70%) and EE (~ 82%). In vitro study showed that effective drug release in an acidic environment could be achieved. Further, we confirmed the activated macrophage could uptake much more FPD/MV/MTX@ZIF-8 than inactivated cells. In vivo biodistribution experiment displayed FPD/MV/MTX@ZIF-8 nanoparticles showed the longest circulation time and best joint targeting. Furthermore, pharmacodynamic experiments confirmed that FPD/MV/MTX@ZIF-8 showed sufficient therapeutic efficacy and safety to explore clinical applications.

**Conclusions:**

This study provides a novel approach for the development of biocompatible drug-encapsulating nanomaterials based on MV-coated metal-organic frameworks for effective RA treatment.

**Graphical Abstract:**

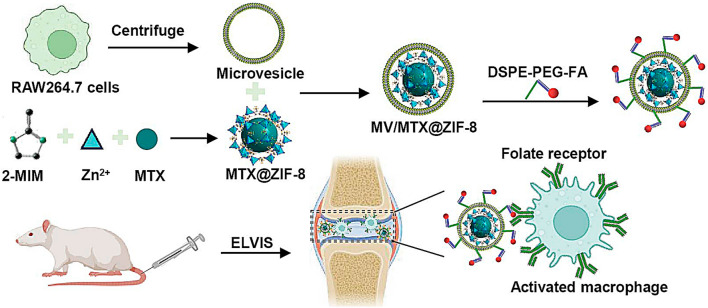

**Supplementary Information:**

The online version contains supplementary material available at 10.1186/s12951-022-01447-0.

## Introduction

Rheumatoid arthritis (RA) is a chronic systemic autoimmune disease characterized by chronic synovitis that causes joint inflammation, synovial hyperplasia, pannus formation, and destruction of bones and cartilage [1, 2]. RA is also often accompanied by persistent arthritis pain, swelling, stiffness, and it may cause serious cardiovascular, pulmonary, psychological, and bone diseases [3, 4]. Disease-modifying anti-rheumatic drugs (DMARDs) can reduce joint damage and relieve disease development [5, 6], thus improving clinical therapy at the early stage of disease [7, 8]. Methotrexate (MTX) has been highlighted as a first-line DMARD for RA treatment [9], as it can inhibit pro-inflammatory cytokine secretion and thereby attenuate inflammatory reactions at lesion sites [10]. However, MTX does not accumulate well at inflamed sites, and long-term administration in high doses leads to severe side effects, including bone marrow suppression, liver and kidney damage, and gastrointestinal dysfunction [11].

Nanomedicines can selectively accumulate at the site of inflammation through the effect “extravasation through leaky vasculature and subsequent inflammatory cell-mediated sequestration” (ELVIS), thereby improving the pharmacokinetic properties and biodistribution of the drug [12]. For instance, nanoparticles encapsulating MTX, including liposomes, hydrogels, nanoemulsions and micelles, enhance the therapeutic efficacy of MTX against RA. However, the drug loading capacity of these drug delivery systems needs to be improved [13, 14].

In recent years, metal–organic frameworks (MOFs) have been used extensively in drug delivery systems due to their large specific surface area, high porosity, adjustable pore size and structure, and hydrophilic cavity design function [15], which allow them to accommodate large amounts of drugs with different physical and chemical properties [16, 17]. Among the most commonly used MOFs is the zeolitic imidazolate framework-8 (ZIF-8), an organic molecular sieve with a zeolite structure, as it can be easily prepared and shows sustained release properties and good biodegradability under acidic conditions [18, 19]. In addition, the zinc ion protects bone and promotes its repair [20–23]. Nevertheless, ZIF-8-based drug carriers are rapidly eliminated by the mononuclear phagocytic system (MPS) and show poor biocompatibility [24]. To increase their therapeutic efficacy, such drug delivery systems can be camouflaged with naturally derived materials to form “core-shell” nanoparticles that persist in circulation and do not elicit strong immune responses in vivo.

Extracellular vesicles with lipid bilayer structures, such as exosomes and microvesicles (MVs), are secreted by mesenchymal stromal cells [25], neural stem cells [26], microglia [27] and other cell types. These vesicles can fuse with the cell membrane, providing a way for the vesicle contents to be transported between cells and for the vesicles to escape phagocytosis by endothelial reticular cells, thereby stabilizing them in the circulation [28, 29]. MVs are small vesicles with a diameter of 200–1000 nm that form directly from the cell membrane of activated cells [30]. MVs can be isolated more easily than exosomes, and they contain membrane-bound proteins from the cells of origin that allow them to bind tightly to target cells [31]. For example, MVs derived from macrophages at sites of inflammation in RA intrinsically target inflamed areas [32]. Negatively charged MVs can also bind to the positively charged surface of ZIF-8 *via* electrostatic and hydrophilic interactions [33], while unsaturated zinc ions on the ZIF-8 nanoparticle surface can coordinate with the P–O bonds of the phospholipid molecules in MVs, stabilizing the nanoparticle [33] (Additional file 1: Fig. S1).

**Scheme 1:**
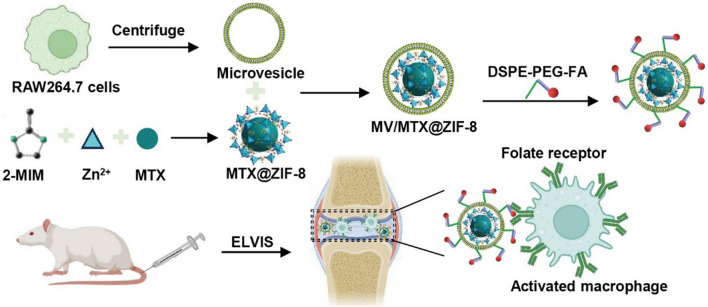
Schematic illustration of the procedure of preparing biomimetic FPD/MV/MTX@ZIF-8 nanoparticles for targeting and intracellular delivery of drugs

Here, we prepared a biomimetic nanoplatform by encapsulating MTX-loaded ZIF-8 (MTX@ZIF-8) into an MV derived from macrophages(Scheme 1). Due to the overexpression of folate receptor β on the surface of activated macrophages [34, 35], the MV/MTX@ZIF-8 nanoparticles were further modified with 1,2-distearoyl-*sn*-glycero-3-phosphoethanolamine-*N*-[folate (polyethylene glycol)-2000] (FPD) to enhance their inflammation-targeting ability. The resulting drug delivery system (FPD/MV/MTX@ZIF-8) exhibited enhanced drug loading capacity, controlled drug release under acidic conditions, good biocompatibility, and prolonged time in circulation [36]. In our previous work, we achieved only 20% dexamethasone loading in exosomes [37], and the DLE was 3.49% for plasmid DNA encoding the anti-inflammatory cytokine interleukin-10 and 9.67% for betamethasone sodium phosphate into biomimetic vector M_2_ exosomes [38], while we achieved much higher loading of 70% with FPD/MV/MTX@ZIF-8.

## Methods

### Materials

MTX and 2-methylimidazole (2-MIM) were provided by Solarbio Science & Technology (Beijing, China); zinc nitrate hexahydrate [Zn(NO_3_)_2_·6H_2_O, ≥ 99%], by Innochem Chemical Reagent Factory (Beijing, China); and FPD, by Xi’an Ruixi Biotechnology Company Ltd. (Xian, China). 3-(4,5-dimethylthiazol-2-yl)-2,5-diphenyltetrazolium bromide (MTT), lipopolysaccharide (LPS), and 4,6-diamidino-2-phenylindole (DAPI) were obtained from Beijing Solarbio Science & Technology. Rhodamine B (Rhm B) and methanol were purchased from Kelong Chemical Reagent Factory (Chengdu, China). The PKH67 fluorescent labeling kit was obtained from Beijing Baiao Laibo Technology (Beijing, China). Rabbit monoclonal antibodies against CD81, CD9, or TSG101, as well as horseradish peroxidase (HRP)-conjugated goat anti-rabbit IgG were obtained from Abcam (Cambridge, UK). Ultrapure water (18.2 MΩ) was prepared using a Milli-Q water purification system (Millipore Co, Shanghai, China).

### Cell lines and animals

Murine macrophage-like RAW264.7 cells (Chinese Academy of Sciences, Shanghai, China) were cultured in Dulbecco’s Modified Eagle Medium (Hyclone, HyClone, Logan, UT, United States, USA) supplemented with 10% fetal bovine serum (FBS) (Gibco, USA) and 1% (v/v) penicillin/streptomycin (Hyclone) at 37 °C in an atmosphere containing 5% CO_2_.

Male Sprague − Dawley rats (140 ± 20 g) were provided by Dashuo Experimental Animal Company (Chengdu, China). The rats were kept under standardized circumstances, and all animal experiments were conducted based on the approval of the Animal Care and Ethics Committee of Southwest Medical University (permit no. 20210223-231).

### Synthesis and characterization of MTX@ZIF-8

For the synthesis of MTX@ZIF-8, 12 mg of MTX and 200 mg of Zn(NO_3_)_2_·6H_2_O were dissolved in 5 mL of methanol. After stirring for 5 min, 10 mL of a MIM (2.0 g) solution in methanol was added, followed by stirring for another 30 min. Next, the solution was centrifuged at 4500×*g* for 5 min to obtain the MTX@ZIF-8 nanoparticles, which were washed at least three times with 10 mL of methanol. Pure ZIF-8 and Rhm B-encapsulated ZIF-8 (Rhm B@ZIF-8) were also prepared as controls using the same method [19, 39].

The morphology of all formulations was observed by transmission electron microscopy (TEM; JEM-1200EX; JEOL Ltd, Tokyo, Japan). The mean particle size and zeta potential were measured by dynamic light scattering (DLS; Malvern Instruments, Worcestershire, UK), and their ultraviolet-visible absorption spectra were recorded on a spectrophotometer (UV-A360, Aoyi, Beijing, China). X-ray powder diffraction (XRD) spectra were obtained with an X’Pert3 Powder diffractometer (D8 Advance, Brucker, Germany) using Cu Kα radiation (2θ = 10–50°). Thermal gravimetric analysis (TGA) was performed on a NETZSCH STA 449 F5/F3 Jupiter instrument (Netzsch, Germany). The surface area of the samples was determined by N_2_ adsorption/desorption at liquid nitrogen temperatures on an ASAP 2460 analyzer (Thermo Fisher Scientific Surfer, ASAP 2460, USA) [36, 40].

### Drug loading and encapsulation efficiency

The encapsulation efficiency (EE) and drug loading efficiency (DLE) of the prepared formulations were determined by high-performance liquid chromatography (HPLC) [36]. The payload of MTX was calculated from the standard calibration curve based on the difference in the amount of MTX between the precipitate collected after centrifugation and the initial solution. DLE and EE were calculated using the following formulas: DLE (%)=(Weight of encapsulated MTX in nanoparticles)/(Total weight of nanoparticles)×100%, EE (%)=(Weight of MTX in nanoparticles)/(Weight of MTX initially added).

### Isolation and characterization of MV, MV/MTX@ZIF-8, and FPD/MV/MTX@ZIF-8

RAW264.7 cells were seeded into a 10-cm dish at 2 × 10^6^ cells/dish and cultured in 7 mL of FBS-free DMEM medium. After two days, the conditioned medium was harvested and centrifuged at 2000×*g* (20 min, 4 °C) to remove cells and debris. The supernatant was then concentrated to about 30% of the original volume at 2400×*g* for 8 min using an ultrafiltration tube (molecular weight cutoff = 10,000 Da; QPTimaMAX-XP Ultra-High, Beckman Coulter, USA). The obtained supernatant was again centrifuged at 16,500×*g* (30 min, 4 °C) and the MVs were collected [41]. After washing once with PBS, the MVs were resuspended in sterile phosphate-buffered saline (PBS) for subsequent experiments.

For the preparation of MV/MTX@ZIF-8, MVs were mixed with 1 mg of MTX@ZIF-8, and the mixture was sonicated for 60 s. The solution was then extruded 10 times through a 200-nm porous polycarbonate membrane, followed by centrifugation at 10,000×*g* for 5 min [42]. Afterward, the collected product and FPD ligands were mixed in a mass ratio of 1:5 and incubated at 37 °C for 2 h. Before use, all preparations were centrifuged at 1000×*g* for 10 min and washed with PBS at least three times [43].

The size distribution and zeta potential of FPD/MV/MTX@ZIF-8 were determined by DLS, and their morphology was examined by TEM. The amount of protein in the purified samples was determined using the bicinchoninic acid (BCA) assay (Beyotime Biotech, Jiangsu, China). In vitro stability of FPD/MV/MTX@ZIF-8 by assessing the changes in size and zeta potential over time. Absorption spectra of the FPD/MV/ZIF-8 and folic acid (FA)were analyzed by UV-Vis spectrophotometry in the range of 190–1100 nm.

### Western blot analysis

The presence of TSG101, CD81, and CD9 on the MV surface was determined by Western blotting [44–46]. The protein concentration was measured using the BCA assay following the manufacturer’s instructions. Briefly, MVs and cell proteins were heated at 100 °C for 5 min. The samples were then fractionated by 10% sodium dodecyl sulfate-polyacrylamide gel electrophoresis and transferred electrophoretically to a polyvinylidene fluoride membrane. The membrane was treated with 5% non-fat dry milk for 2 h in washing medium to block non-specific binding sites, then incubated with primary antibodies against TSG101, CD81, or CD9 (1:1000; Abcam, UK) [38] at 4 °C overnight. After washing with TBST, the membrane was incubated with horseradish peroxidase-conjugated anti-rabbit secondary antibody (1:1000; Beyotime Biotechnology) at room temperature for 1 h. The membrane was finally soaked in chemiluminescent HRP substrate (Solarbio Biotechnology Company), and chemiluminescence was measured with a ChemiDoc instrument (Bio-Rad, Hercules, CA, USA).

### Cumulative drug release

The cumulative drug release of free MTX, MTX@ZIF-8, MV/MTX@ZIF-8, and FPD/MV/MTX@ZIF-8 was determined by a dialysis method [19]. Briefly, 1 mL of each formulation was sealed in a dialysis bag (molecular weight cutoff = 14,000 Da) and incubated in 50 mL of PBS (pH 7.4 and 5) at 37 °C for 24 h under stirring. At predetermined time points, 200 µL of the release medium was collected and replaced with an equal volume of fresh medium. The concentration of MTX was determined by HPLC [39], and the cumulative amount released was calculated using the formula: Drug released (%) = (amount of drug in release medium/amount of drug loaded into nanoparticles) × 100%. The experiment was performed in triplicate.

### In vitro cytotoxicity

The in vitro cytotoxicity of free MTX, pure ZIF-8, MTX@ZIF-8, MV/MTX@ZIF-8, FPD/MV/MTX@ZIF-8, MV/ZIF-8 or FPD/MV/ZIF-8 was examined in RAW264.7 cells using the MTT method [47]. First, cells were seeded in 96-well plates (1 × 10^4^/well) and incubated in 200 µL of cell medium overnight at 37 °C in an atmosphere containing 5% CO_2_. Next, the culture medium was removed and different concentrations of free MTX, MTX@ZIF-8, MV/MTX@ZIF-8, FPD/MV/MTX@ZIF-8, MV/ZIF-8 or FPD/MV/ZIF-8 were added. After incubation for 24 h, MTT solution (5 mg/mL) was added to each well, followed by incubation at 37 °C for 4 h. Afterward, 150 µL of dimethyl sulfoxide was added to each well to solubilize the formazan crystals. Absorbance at 490 nm was measured using a microplate reader (Synergy H1, Biotek, Vermont, USA). Cells cultured in nanoparticle-free medium were used as controls. All cytotoxicity experiments were performed three times.

Replace the MTT solution in the experimental steps for MTT with CCK-8 solution for CCK-8 assay. In brief, 10 µL CCK-8 was added into each well and then further cultured RAW264.7 cells for 2 h. The absorbance at 450 nm was measured with a microplate reader.

For Calcein- AM /PI staining, RAW264.7 cells were inoculated in 12-well plates and incubated overnight, followed by co-incubation with the different preparations for 24 h. 5 µL Calcein-AM solution (2 mM) and 15 µL PI solution (1.5 mM) were added to the 5 mL of 1 × Assay Buffer to obtain the staining working solution. Then, added staining working solution to the cells in each well and incubated at 37 °C for 15 min. Photographs were taken by employing the fluorescence microscope.

### Uptake of nanoparticles by cells

MTX@ZIF-8, MV/MTX@ZIF-8, and FPD/MV/MTX@ZIF-8 were labeled with Rhm B and PKH67 according to the manufacturer’s protocol. Resting RAW264.7 cells or RAW264.7 cells stimulated for 24 h with LPS at a final concentration of 10 µg/mL were then incubated with the labeled formulations for 1 h at 37 °C [48]. After staining the cell nuclei with DAPI, endocytosis was observed by confocal laser scanning microscopy (Leica Microsystems, Wetzlar, Germany) [37]. Uptake of the labeled formulations was also observed by flow cytometry using a Verse cytometer (BD Biosciences, Franklin Lakes, NJ, USA) after stimulating RAW264.7 cells for 24 h with LPS. To determine whether folate receptor β can interact directly with FPD ligands, activated RAW264.7 cells were pretreated with folic acid (FA) solution (100 µg/ml) to saturate folate receptor β before incubation with FPD/MV/MTX@ZIF-8 NPs.

### Anti-inflammatory effects of nanoparticles against LPS-activated RAW264.7 cells

RAW264.7 cells were seeded into a 24-well plate (5 × 10^5^ cells/well) and incubated with LPS at a final concentration of 10 µg/mL at 37 °C for 24 h. The culture medium was then replaced with fresh medium containing free MTX, MTX@ZIF-8, MV/MTX@ZIF-8, FPD/MV/MTX@ZIF-8, MV/ZIF-8 or FPD/MV/ZIF-8 at a final MTX concentration of 20 µg/mL. Cells cultured in PBS-containing medium were used as negative controls. After incubation at 37 °C for 24 h, the culture medium was collected and centrifuged at 2000×*g* for 5 min. The levels of tumor necrosis factor-α (TNF-α), interleukin (IL)-1β, and IL-10 in the supernatant were determined using commercial enzyme-linked immunosorbent assays (ELISAs, Thermo Fisher, Austria) following the manufacturer’s instructions [49].

### Hemolysis assay

Fresh blood samples collected from healthy mice were centrifuged at 800×*g* for 10 min, and the red blood cells (RBCs) were isolated. After washing with saline at least three times, a 2% solution of RBCs in saline was prepared. Free MTX, MTX@ZIF-8, MV/MTX@ZIF-8, or FPD/MV/MTX@ZIF-8 in saline were then mixed with an identical volume of 2% RBCs, incubated at 37 °C for 3 h, and centrifuged at 800×*g* for 10 min. Saline was used as a negative control and deionized water as a positive control. The absorbance of the supernatant at 545 nm was measured using a microplate reader. The hemolysis percentage was calculated as follows [36]: (Sample absorbance – Negative control absorbance)/(Positive control absorbance – Negative control absorbance)×100%.

### Rat model of collagen-induced arthritis (CIA)

To establish the CIA rat model, bovine type II collagen was thoroughly emulsified with an equal volume of complete Freund’s adjuvant (5 mg/mL; Chondrex, USA) by vortex mixing, and 100 µL of the emulsion was administered intradermally to the base of the rat tail. After seven days, rats were administered an intradermal booster injection of type II collagen with an equal volume of incomplete Freund’s adjuvant. RA severity was evaluated by rating the paw swelling according to the following scale [37]: 0, no signs of swelling; 1, mild inflammation and swelling of individual toes; 2, moderate inflammation and swelling of all toes; 3, severe swelling of the entire paw; and 4, maximum swelling of the limb. The scores of each paw were summed to calculate the total arthritis index score for the animal.

### Biodistribution and pharmacokinetics

The biodistribution of nanoparticles was observed using near-infrared fluorescence imaging. CIA rats were intravenously administered with free Cy5, Cy5 encapsulated in ZIF-8 (Cy5@ZIF-8), MV/Cy5@ZIF-8, or FPD/MV/Cy5@ZIF-8. In each case, the Cy5 dose was 5 mg/rat. At 1, 12, and 24 h post-injection, rats were sacrificed, and their major organs were collected. The fluorescence intensity in the collected tissues was analyzed using an IVIS Spectrum system (Caliper, Hopkinton, MA, USA).

CIA rats were also randomly divided into four groups (n = 3 per group) and intravenously injected once with free MTX, MTX@ZIF-8, MV/MTX@ZIF-8, or FPD/MV/MTX@ZIF-8. In each case, the MTX dose was 1 mg per kg. At 0.25, 0.5, 1, 2, 4, and 8 h post-injection, blood was collected for quantitative analyses of pharmacokinetics. At 0.25, 1, 4 and 8 h post-injection, the rats were sacrificed and their heart, liver, spleen, lung, kidneys, entire hind limbs were collected for quantitative analyses of biodistribution.

### Weight, ankle diameter, paw thickness, foot volume, and articular index score in CIA rats

On day 16 after collagen induction, CIA rats were also randomly divided into seven groups (n = 3 per group) which were injected via the tail vein with saline, ZIF-8, free MTX, MTX@ZIF-8, MV/MTX@ZIF-8, or FPD/MV/MTX@ZIF-8 (MTX dose = 1 mg/kg) once every three days for a total of six times. Healthy rats were used as a control. Arthritic index scores were determined for each limb as described in “Rat model of collagen-induced arthritis (CIA)” section.  Body weight, ankle diameter, right hind paw thickness, and foot volume were also measured during treatment every three days. Foot volume was measured using the drainage method [37].

### Micro-computed tomography (micro-CT) of articular bone

On day 34 after collagen induction, rats in each treatment group were euthanized, and their hind limbs were collected. After removing the muscles, the hind limbs were fixed in formalin overnight. The microstructure of each limb was then analyzed using an Inveon positron emission tomography/computed tomography system (Siemens, Siemens, Erlangen, Germany, Germany). The bone mineral density (BMD), ratio of bone surface area to bone volume (BS/BV), ratio of bone volume to tissue volume (BV/TV), trabecular number (Tb.N), trabecular separation (Tb.Sp), and trabecular thickness (Tb.Th) in regions of interest were calculated using Siemens Inveon Research Workplace software 4.2 [1].

### Pro-inflammatory cytokines in serum

Blood samples were collected from rats on day 34 after CIA induction, and serum levels of the pro-inflammatory cytokines TNF-α and IL-1β were measured using a commercial ELISA (see section Anti-inflammatory effects of nanoparticles against LPS-activated RAW264.7 cells) according to the manufacturer’s instructions.

### Histological evaluation of joint tissue

Joint tissue samples were decalcified with 15% ethylenediaminetetraacetic acid at neutral pH. Decalcification was considered complete when there was no resistance to acupuncture. The decalcified tissue was then embedded in paraffin and cut into thin sections, which were stained with hematoxylin and eosin (H&E), safranin O or toluidine blue. The histological score was determined by rating cellular infiltrates and cartilage erosion on a scale [50] from 0 to 3, where 0 = no symptoms, 1 = mild symptoms (1–10% cellular infiltrates and cartilage erosion), 2 = moderate symptoms (11–50%), and 3 = severe symptoms (51–100%).

### In vivo safety evaluation

After the whole treatment, serum of each group was collected on day 34. The levels of aspartate transaminase (AST) and alanine transaminase (ALT) in serum collected from different treatment groups were determined using an automatic biochemical analyzer (Mindray BS-240, Shenzhen, China).

### Statistical analysis

All data were expressed as mean ± standard deviation (SD). Statistical analysis was performed using GraphPad Prism 7 (GraphPad Software, La Jolla, CA, USA). One- or two-way analysis of variance was used to assess inter-group differences for significance, and differences associated with *P* < 0.05 were considered significant.

## Results

### Characterization of ZIF-8 and MTX@ZIF-8

**Fig. 1:**
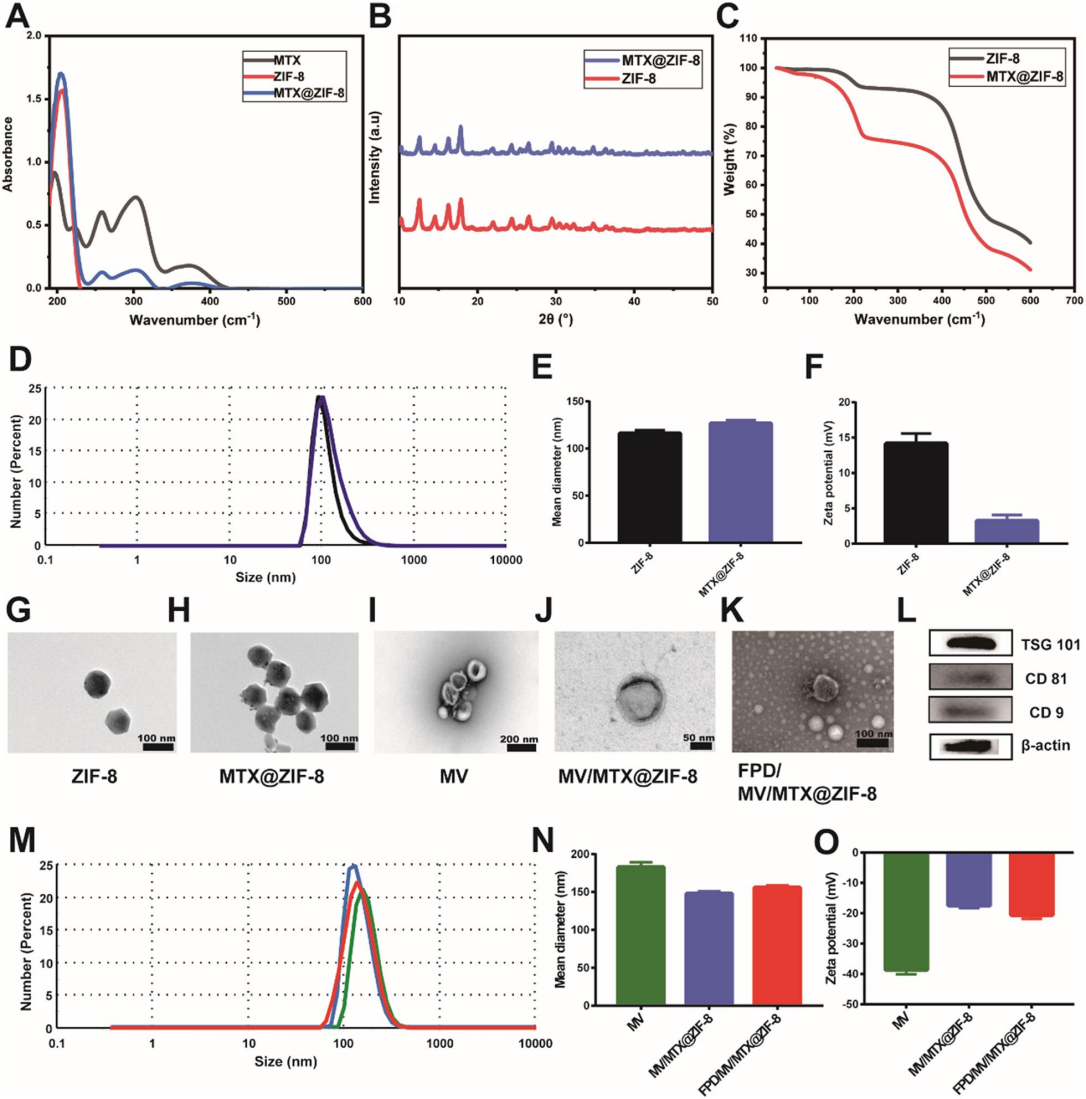
Physicochemical characterization of different formulations. **A** Ultraviolet-visible spectra of free MTX, bare ZIF-8, and MTX@ZIF-8. **B** X-ray diffraction patterns. **C** TGA absorption spectra of ZIF-8 and MTX@ZIF-8. **D** Size distribution, **E** size, and **F** zeta potential of ZIF-8 and MTX@ZIF-8. **G–K** Transmission electron micrographs of different formulations. **L** Representative Western blots for TSG101, CD81 and CD9. **M** Size distribution, **N** mean diameter, and **O** zeta potential of MVs, MV/MTX@ZIF-8, and FPD/MV/MTX@ZIF-8. FPD: 1,2-distearoyl-*sn*-glycero-3-phosphoethanolamine-*N*-[folate (polyethylene glycol)-2000; MTX: methotrexate; MV: microvesicle; ZIF-8: zeolitic imidazolate framework-8

The encapsulation of MTX in ZIF-8 was verified by UV-Vis spectroscopy (Fig. 1A). MTX and MTX@ZIF-8 showed the same characteristic absorption peaks at 258 and 302 nm [48], indicating the successful loading of MTX into the ZIF-8 structure. Moreover, MTX@ZIF-8 showed a similar XRD pattern to ZIF-8, suggesting the negligible effect of MTX on the crystal structure of ZIF-8 (Fig. 1B). TGA measurements revealed that ZIF-8 and MTX@ZIF-8 had good thermal stability, with little weight loss at temperatures up to 400 °C (Fig. 1C). According to the Bruner-Emmett-Teller (BET) analysis (Additional file 1: Fig. S2A, B), all samples displayed type-I adsorption-desorption isotherms. The BET surface and pore volume of MTX@ZIF-8 decreased to 686.94 m^2^/g and 0.399 cm^3^/g in comparison with that of ZIF-8 (1208.87 m^2^/g and 0.735 cm^3^/g), as MTX was filled in the pores due to its small size, which indicated that MTX was encapsulated into the crystal of ZIF-8. DLS measurements also showed that the mean diameter of the MTX@ZIF-8 nanoparticles (126.6 ± 3.51 nm) was slightly higher than that of bare ZIF-8 (116 ± 3.61 nm), while their zeta potential (3.24 ± 0.83 mV) was considerably lower than that of pure ZIF-8 (14.17 ± 1.42 mV) (Fig. 1D–F). Interestingly, the roughly spherical shape of bare ZIF-8 changed only slightly after drug loading (Fig. 1G, H). The EE and DLE of MTX@ZIF-8 were 82.77 ± 3.36% and 70.03 ± 2.86%, respectively.

### Characterization of MVs, MV/MTX@ZIF-8 and FPD/MV/MTX@ZIF-8

Based on protein content measurements, 16.49 ± 3.44 µg of MVs could be extracted from 1 mL of culture medium. TEM analysis showed that MVs were round-shaped particles surrounded by a membrane, while MV/MTX@ZIF-8 and FPD/MV/MTX@ZIF-8 had a clear core-shell structure (Fig. 1I–K). DLS measurements also indicated that the hydrodynamic diameter of MTX@ZIF-8 nanoparticles increased after coating with MVs (from 126.6 ± 3.51 to 147.7 ± 3.21 nm), approximately consistent with the thickness reported for the lipid bilayer of macrophage-derived MVs (~ 8 nm) [51]. In contrast to MTX@ZIF-8, the zeta potential of MV/MTX@ZIF-8 was negative, probably due to charge shielding by the MV coating [33] (Fig. 1M–O). Meanwhile, both the size and zeta potential changes of the FPD/MV/MTX@ZIF-8 nanoparticles were not significant, which indicated their stability over 72 h (Additional file 1: Fig. S3), and absorption peaks at 285 nm for both FPD/MV/ZIF-8 and FA (Additional file 1: Fig. S4), indicating successful integration of the folate ligand in the nanosystem. Finally, western blot experiments showed that the MVs contained the expected marker proteins TSG101, CD81, and CD9 (Fig. 1L).

### In vitro cumulative release of MTX

**Fig. 2:**
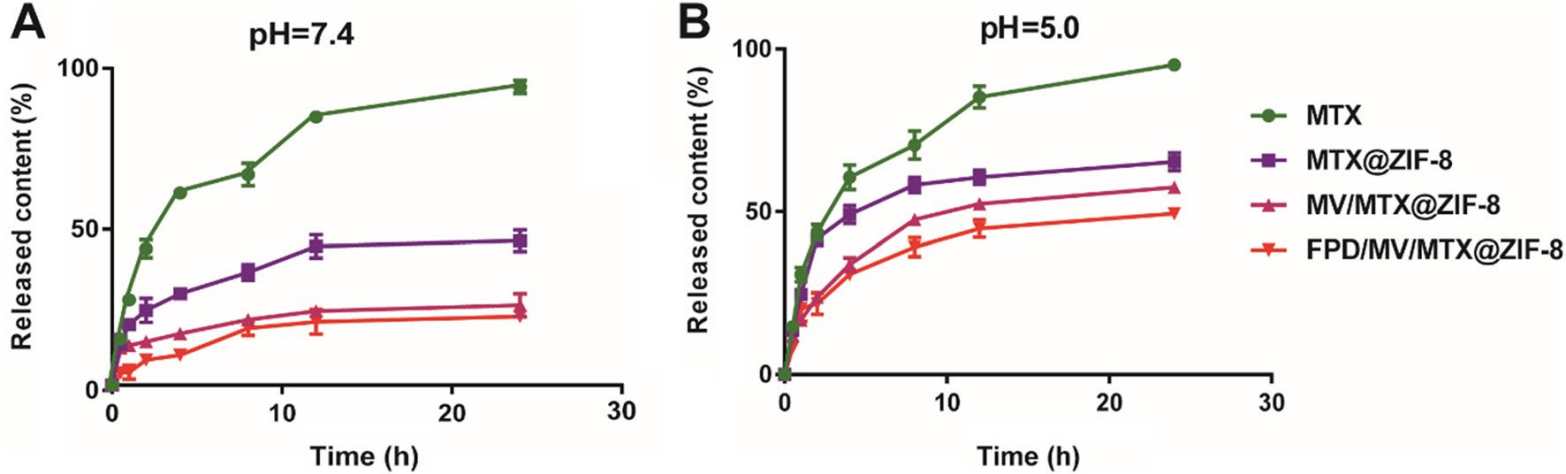
In vitro cumulative release of various formulations at (**A**) pH 7.4 and (**B**) pH 5.0. Data are shown as mean ± SD (n = 3). FPD: 1,2-distearoyl-*sn*-glycero-3-phosphoethanolamine-*N*-[folate (polyethylene glycol)-2000; MTX: methotrexate; MV: microvesicle; ZIF-8: zeolitic imidazolate framework-8

To investigate the cumulative release of MTX from the different formulations in vitro, the drug concentration in the release medium was determined by HPLC. At pH 7.4, over 90% of free MTX was released within 24 h, while only ~ 50% of MTX@ZIF-8 was released over the same period (Fig. 2A). In contrast, the cumulative release of MTX from MV/MTX@ZIF-8 and FPD/MV/MTX@ZIF-8 nanoparticles within 24 h was only 25% and 20%, respectively. This suggests that the MV coating contributed to the sustained release of MTX and could guarantee minimal drug leakage during blood circulation.

At pH 5.0, MTX@ZIF-8 released about 50% of the drug in only 4 h, implying that ZIF-8 can release MTX in a pH-responsive manner. TEM also confirmed that MTX@ZIF-8 degraded in a few hours under acidic conditions (Additional file 1: Fig. S5). This rapid release was attributed to facile hydrolysis of the metal-ligand bond in MOFs under weak acidic conditions, which leads to protonated ligands [33]. Similarly, MV/MTX@ZIF-8 and FPD/MV/MTX@ZIF-8 released 57% and 49% of the drug, respectively, in 4 h; this was more than twice the rates observed at pH 7.4, suggesting that the ZIF-8 structure degraded more easily at pH 5.0 due to the dissociation of the coordination bond between zinc and imidazole [52]. The faster drug release could also reflect the proton-sponge effect (Additional file 1: Fig. S6). After adsorption, the designed pH-responsive nanoparticles can release organic ligands of imidazole derivatives, which can buffer the protonation of the imidazole ring. Protons accumulate along with their counterions in the endosome, stimulating the entry of water from the cytoplasm to balance the high osmotic pressure in the endosome. Swelling of the endosome in the presence of ligands eventually leads to endosome rupture and release of guest molecules [33].

### Nanoparticle cytotoxicity

Free MTX and MTX@ZIF-8 showed high cytotoxicity to RAW264.7 cells even at 5 µg/mL, implying that the MTX@ZIF-8 nanoparticles may be non-biocompatibility, consistent with a previous report [48]. In contrast, the viability of cells treated with MV/MTX@ZIF-8, or FPD/MV/MTX@ZIF-8 was higher even at 25 µg/mL (Additional file 1: Fig. S7), suggesting that the MV coating improved biocompatibility and selectivity toward macrophages. Meanwhile, we found that MTT and CCK-8 results were generally consistent (Additional file 1: Fig. S7A,B), MTX and MTX@ZIF-8 were highly cytotoxic to RAW264.7 cells, compared to MV/MTX@ZIF-8 or FPD/MV/MTX@ZIF-8 treated cells. In addition, FPD/MV/MTX@ZIF-8 showed safer than MTX, which could be seen from the Calcein-AM/PI staining results (Additional file 1: Fig. S7C).

### Cellular uptake of nanoparticles by RAW264.7 cells

**Fig. 3:**
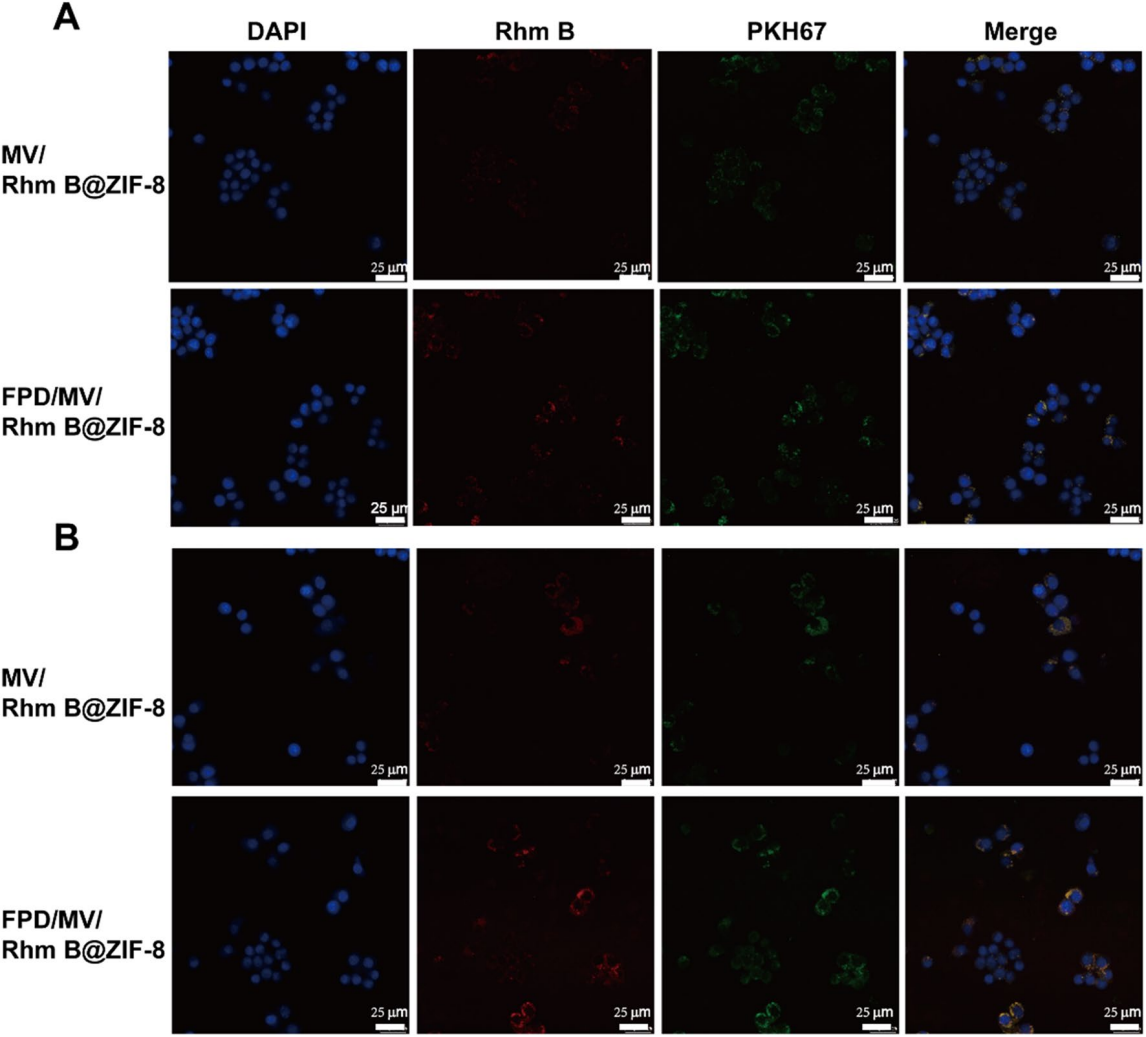
Cellular uptake of rhodamine B (Rhm B)-labeled formulations by (**A**) non-activated RAW264.7 and (**B**) LPS-activated RAW264.7 cells, as determined by confocal microscopy. Scale bar: 25 μm. DAPI: 4,6-diamidino-2-phenylindole; FPD: 1,2-distearoyl-*sn*-glycero-3-phosphoethanolamine-*N*-[folate (polyethylene glycol)-2000; LPS: lipopolysaccharide; MV: microvesicle; ZIF-8: zeolitic imidazolate framework-8

**Fig. 4:**
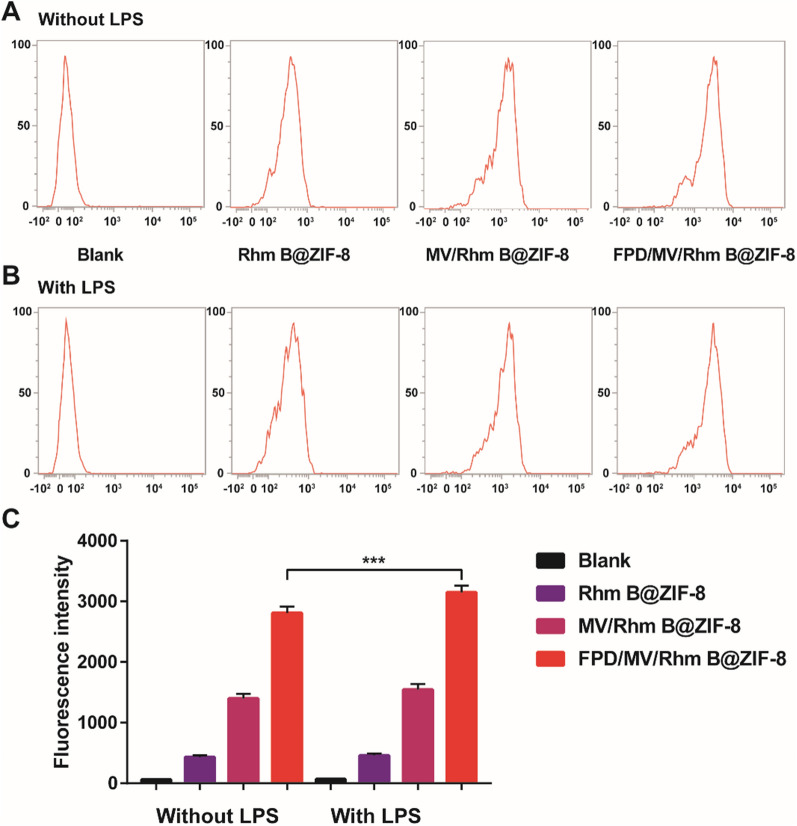
Cellular uptake of rhodamine B (Rhm B)-labeled formulations by **A** Non-activated and **B** LPS-activated RAW264.7 cells, as determined by flow cytometry. The horizontal axis indicates fluorescence intensity and the vertical axis cell indices counts. **C** Uptake rate of different preparations, as determined by flow cytometry. Data are shown as mean ± SD (n = 3). ^***^P < 0.001. FPD: 1,2-distearoyl-*sn*-glycero-3-phosphoethanolamine-*N*-[folate (polyethylene glycol)-2000; LPS: lipopolysaccharide; MV: microvesicle; ZIF-8: zeolitic imidazolate framework-8

To evaluate the cellular uptake of nanoparticles by RAW264.7 cells, we prepared formulations encapsulating the fluorescent probe Rhm B instead of MTX. LPS-activated cells took up more Rhm B@ZIF-8, MV/Rhm B@ZIF-8, and FPD/MV/Rhm B@ZIF-8 than non-activated cells (Fig. 3 and Additional file 1: Fig. S8). Interestingly, FPD/MV/Rhm B@ZIF-8 showed the highest uptake irrespective of whether the cells were activated or not, indicating the high affinity of this formulation for the target cells. These results were confirmed by flow cytometry, which showed that FPD/MV/Rhm B@ZIF-8 was taken up more than other formulations (Fig. 4). Meanwhile, we found that FA pretreatment resulted in an almost 8-fold reduction in FPD/MV/MTX@ZIF-8 NPs uptake (Additional file 1: Fig. S9), which demonstrated that FPD could specifically target folate receptor β on activated RAW264.7 cells and promote the uptake of FPD/MV/MTX@ZIF-8 NPs by activated RAW264.7 cells.

### In vitro anti-inflammatory activity

To evaluate the anti-inflammatory activity of the prepared formulations in vitro, we measured the levels of TNF-α, IL-1β, and IL-10 in LPS-activated RAW264.7 cells treated with eight formulations. FPD/MV/MTX@ZIF-8 showed the strongest inhibitory effect on TNF-α expression (Additional file 1: Fig. S10A), while it significantly upregulated IL-10 (Additional file 1: Fig. S10C). FPD/MV/MTX@ZIF-8 and MV/MTX@ZIF-8 also significantly inhibited IL-1β secretion (Additional file 1: Fig. S10B), suggesting that the developed drug delivery system has good anti-inflammatory efficacy and enhanced targeting ability owing to the MV coating. Notably, MV/ZIF-8 and FPD/MV/ZIF-8 groups had no effect on cellular secretion of inflammatory factors. Furthermore, all samples caused average hemolysis below 5% (Additional file 1: Fig. S11), indicating good biocompatibility.

### Nanoparticle biodistribution and pharmacokinetics

To evaluate the nanoparticle biodistribution in CIA rats, we prepared formulations encapsulating the fluorescent probe Cy5 and observed their behavior at 1, 12, and 24 h post-injection by near-infrared fluorescence imaging. MV/Cy5@ZIF-8 nanoparticles showed higher fluorescence intensity than free Cy5 and Cy5@ZIF-8 at three time points. FPD/MV/Cy5@ZIF-8 showed the strongest fluorescence intensity among the tested groups (Additional file 1: Fig. S12A), suggesting that the FPD modification endows nanoparticles with greater, longer-lasting inflammation-targeting ability. Semi-quantitation of the fluorescence intensity in ankle joints further confirmed that FPD/MV/Cy5@ZIF-8 led to the strongest fluorescence (Additional file 1: Fig. S12B), suggesting that the FPD modification can greatly enhance drug accumulation at the inflamed sites.

**Fig. 5:**
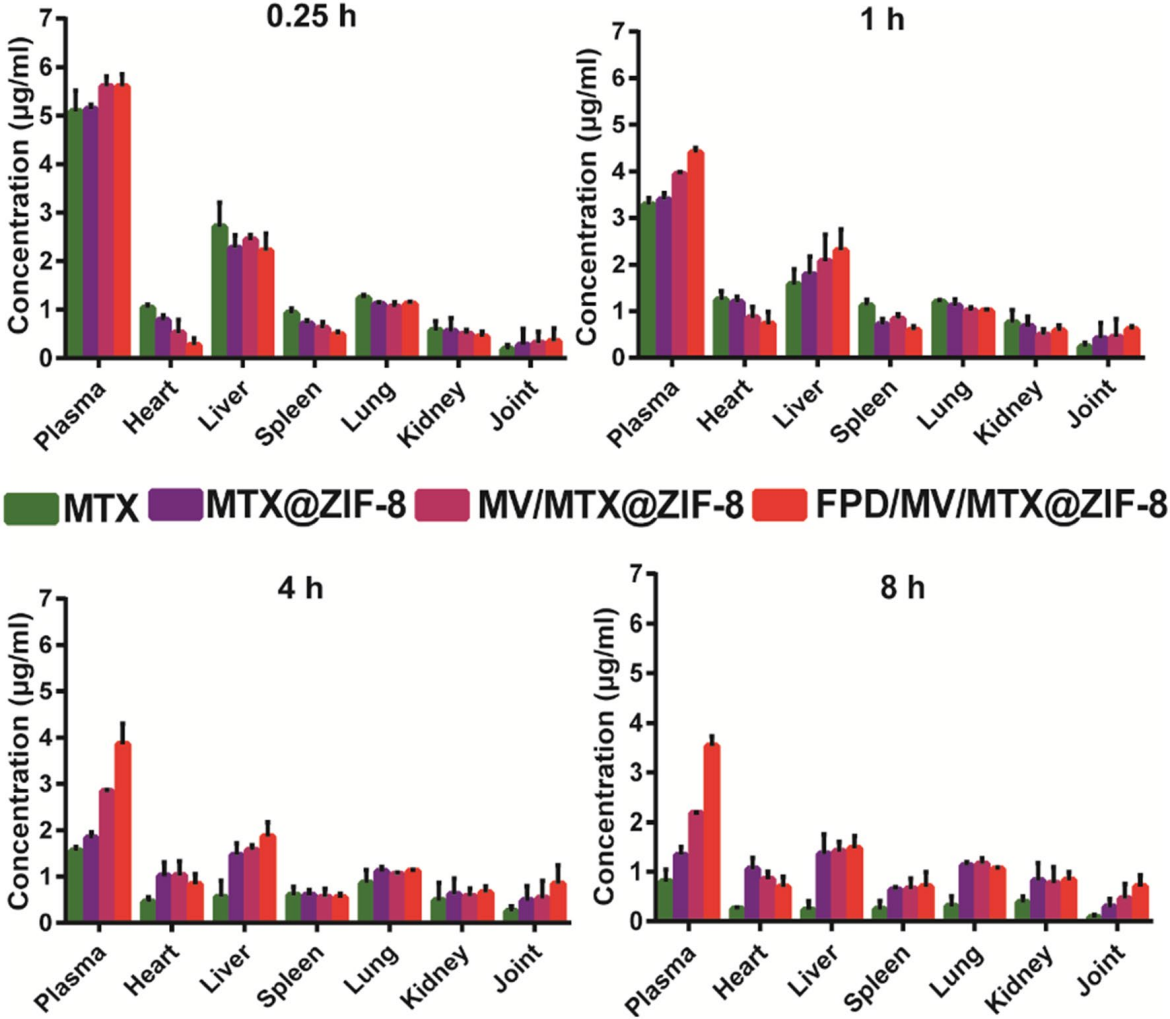
Concentration of methotrexate (MTX) in the tissues of rats with collagen-induced arthritis after tail vein injection of different formulations. Data are shown as mean ± SD (n = 3). FPD: 1,2-distearoyl-*sn*-glycero-3-phosphoethanolamine-*N*-[folate (polyethylene glycol)-2000; MV: microvesicle; ZIF-8: zeolitic imidazolate framework-8

To evaluate the biodistribution of MTX-based preparations, CIA rats were intravenously injected with free MTX, MTX@ZIF-8, MV/MTX@ZIF-8, and FPD/MV/MTX@ZIF-8, and the drug concentration in the tissues collected at different time points was determined by HPLC. Free MTX accumulated mainly in the liver and was rapidly excreted from the body within 8 h. In contrast, MV/MTX@ZIF-8 showed longer circulation time and better distribution of MTX in the joints. Among the examined formulations, FPD/MV/MTX@ZIF-8 nanoparticles showed the longest circulation time and best joint targeting (Fig. 5), confirming that the FPD modification can greatly enhance drug accumulation in inflamed joints.

Further experiments revealed that FPD/MV/MTX@ZIF-8 had the longest blood circulation time, followed by MV/MTX@ZIF-8 and MTX@ZIF-8 (Additional file 1: Fig. S13A). This difference was attributed to the synergistic effect of the MV coating and the PEG chain, which enabled the recognition of the nanoparticles as endogenous vesicles. In addition, the plasma concentration of MTX was significantly higher in the FPD/MV/MTX@ZIF-8 group than in the MTX@ZIF-8 and MV/MTX@ZIF-8 groups, indicating that FPD/MV/MTX@ZIF-8 was cleared in vivo much more slowly than other formulations. Pharmacokinetic studies also showed that the FPD/MV/MTX@ZIF-8 nanoparticles had the longest half-life among the four preparations as well as excellent bioavailability (Additional file 1: Fig. S13B).

### Therapeutic efficacy in vivo

**Fig. 6:**
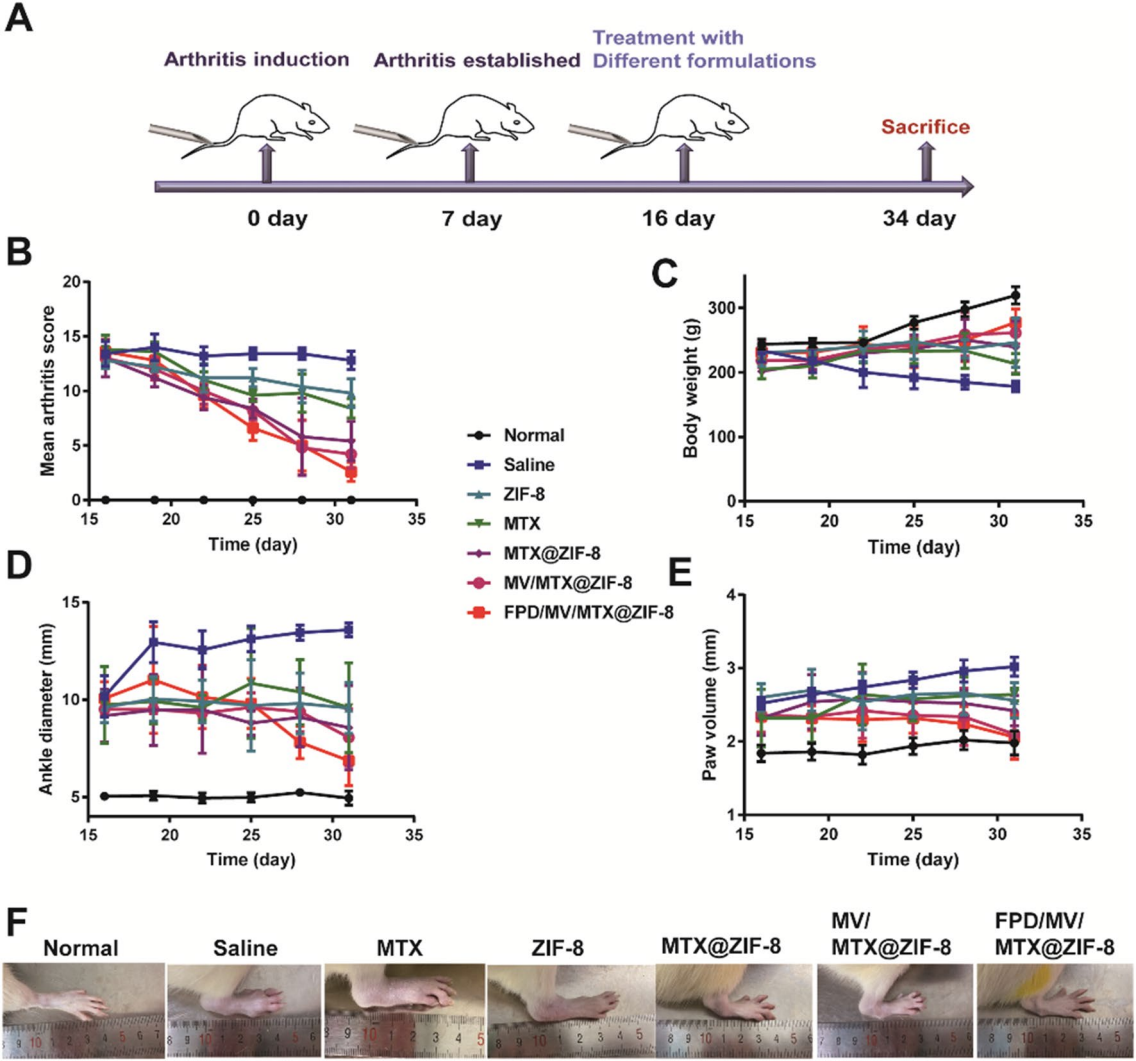
Therapeutic effect of methotrexate (MTX)-based formulations in rats with collagen-induced arthritis (CIA). **A** Schematic illustration of the treatment process. **B–E** Changes in **B** mean articular score, **C** body weight, **D** ankle diameter, and **E** paw volume over time. **F** Photographs of hind limbs of CIA rats treated with various MTX formulations. Data are shown as mean ± SD (n = 3). FPD: 1,2-distearoyl-*sn*-glycero-3-phosphoethanolamine-*N*-[folate (polyethylene glycol)-2000; MV: microvesicle; ZIF-8: zeolitic imidazolate framework-8

At 14 days after arthritis induction, animals showed mild swelling and erythema, which developed into severe swelling and erythema in the hind limbs within two days. The ankle size also reached a maximum, and moderate inflammation was observed in the front limbs. On day 16, the rats were randomly divided into seven groups and intravenously administered different formulations once every three days for six times (Fig. 6A). On day 34 after collagen induction, animals in the FPD/MV/MTX@ZIF-8 group showed significantly lower arthritis progression than other treatment groups in terms of articular index score, body weight, ankle diameter, paw volume, and paw thickness (Fig. 6B–E and Additional file 1: Fig. S14).

To confirm the effects of each formulation, hind limbs of CIA rats were photographed (Fig. 6F). Saline-treated animals showed severe and extensive joint swelling and deformation compared to the normal group. These symptoms were only slightly reduced in the free MTX group, whereas the edema nearly disappeared in the FPD/MV/MTX@ZIF-8 group, which showed no significant difference from the normal group. MV/MTX@ZIF-8 nanoparticles also significantly improved swelling, but their effect was weaker than that of FPD/MV/MTX@ZIF-8, indicating the high efficacy of the FPD-modified nanoplatform.

### Pro-inflammatory cytokines in serum

CIA rats showed increased serum levels of TNF-α and IL-1β. After treatment with free MTX and bare ZIF-8, the levels of TNF-α and IL-1β increased further, whereas FPD/MV/MTX@ZIF-8 and MV/MTX@ZIF-8 significantly reduced the levels of pro-inflammatory cytokines (Additional file 1: Fig. S15). Among the tested preparations, FPD-modified nanoparticles showed the best anti-inflammatory effect.

### Micro-CT analysis of articular bone

**Fig. 7:**
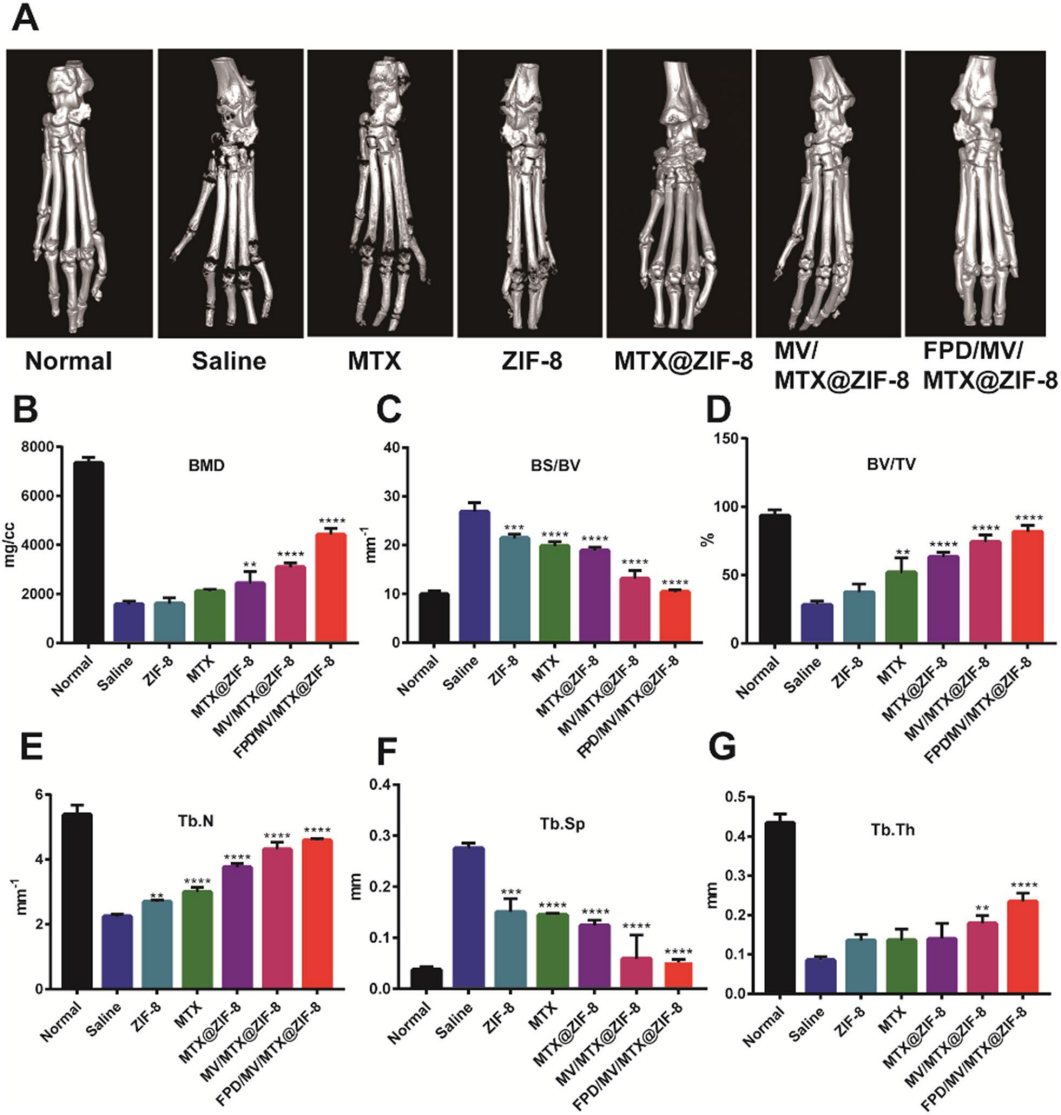
Micro-computed tomography of the hind limbs of rats with collagen-induced arthritis after treatment with different formulations. **A** Representative 3D reconstructed images from each treatment group. **B** Bone mineral density (BMD). **C** Ratio of bone surface area to bone volume (BS/BV). **D** Ratio of bone volume to tissue volume (BV/TV). **E** Trabecular number (Tb.N). **F** Trabecular separation (Tb.Sp). **G** Trabecular thickness (Tb.Th). Data are shown as mean ± SD (n = 3). ^*^P < 0.05, ^**^P < 0.01, ^***^P < 0.001, ^****^P < 0.0001 vs. saline group. FPD: 1,2-distearoyl-*sn*-glycero-3-phosphoethanolamine-*N*-[folate (polyethylene glycol)-2000; MTX: methotrexate; MV: microvesicle; ZIF-8: zeolitic imidazolate framework-8

The efficacy of the developed nanoparticles was also assessed using micro-CT to observe the hind limbs of CIA rats after administration of different formulations (Fig. 7A). Saline-treated rats showed the most severe injury with extensive bone erosion in the ankle and toe joints, whereas FPD/MV/MTX@ZIF-8 completely alleviated joint damage: quantitative analysis of the calcaneal region of interest showed that FPD/MV/MTX@ZIF-8 successfully maintained bone mass in terms of BMD, BS/BV, BV/TV, Tb.N, Tb.Sp, and Tb.Th (Fig. 7B–G), giving values close to those of healthy controls and performing significantly better than the other MTX-based formulations.

### Histological analysis of joint tissue

**Fig. 8:**
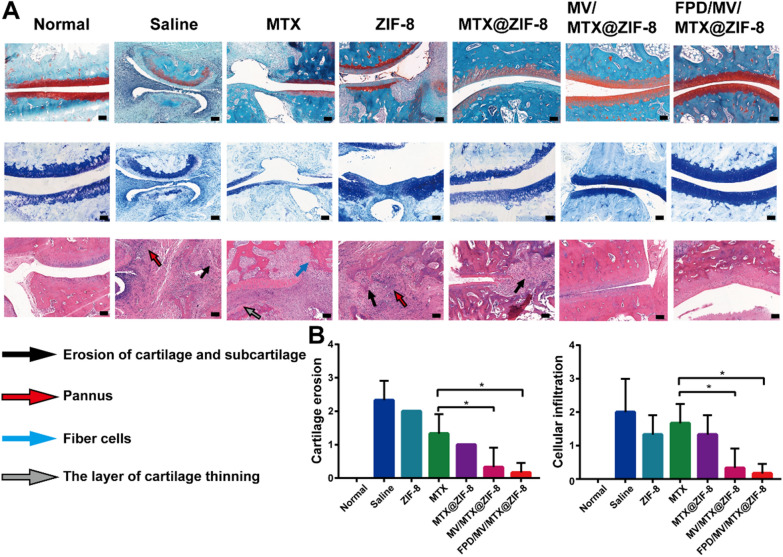
**A** Histopathological evaluation of ankle joints of rats with collagen-induced arthritis after treatment with different formulations using safranin O, toluidine blue, and hematoxylin-eosin staining (100×). Scale bar: 100 μm. **B** Histological scores of cartilage erosion and cellular infiltration. Data are shown as mean ± SD (n = 3). ^*^P < 0.05. FPD: 1,2-distearoyl-*sn*-glycero-3-phosphoethanolamine-*N*-[folate (polyethylene glycol)-2000; MTX: methotrexate; MV: microvesicle; ZIF-8: zeolitic imidazolate framework-8

To further demonstrate that FPD/MV/MTX@ZIF-8 can control inflammation and reduce cartilage destruction, the rat ankle joints were histologically analyzed by H&E, safranin O, and toluidine blue staining. The saline group showed substantial synovial cell proliferation, moderate to severe infiltration of lymphocytes and neutrophils, an increased number of macrophages, and extensive fibrous tissue proliferation. MV/MTX@ZIF-8 and FPD/MV/MTX@ZIF-8 significantly reduced synovial inflammation and cartilage loss, while FPD/MV/MTX@ZIF-8 led to a larger positive area of safranin O and toluidine blue that was similar to healthy controls (Fig. 8A and Additional file 1: Fig. S16). These results suggest that the developed nanoplatform can effectively reduce synovial inflammation and cartilage destruction in CIA rats.

These observations were confirmed by the histopathological evaluation of ankle joints using histological scores (Fig. 8B). Saline-treated CIA rats showed severe cartilage erosion and cellular infiltration with grade > 2. Rats in the MTX group also showed moderate cartilage erosion and cellular infiltration with a grade of ~ 2, whereas the MV/MTX@ZIF-8 and FPD/MV/MTX@ZIF-8 groups showed minimal cartilage erosion and cellular infiltration with grade < 1.

### Hepatotoxicity of nanoparticles in CIA rats

To examine the hepatotoxicity of the developed nanoparticles in CIA rats, levels of AST and ALT in serum were determined. Both levels were significantly higher in the MTX and MTX@ZIF-8 groups than in the normal group, suggesting that these formulations can cause severe liver damage. In contrast, both levels were significantly lower in the FPD/MV/MTX@ZIF-8 group, indicating that the FPD-modified nanoparticles can significantly reduce the hepatotoxicity of MTX (Additional file 1: Fig. S17).

## Discussion

RA is a chronic inflammatory disease in which large numbers of monocytes and macrophages accumulate and infiltrate at the site of inflammation, further promoting inflammation and degrading extracellular matrix [53, 54]. Although several drugs have been developed for RA treatment, their accumulation at sites of inflammation is low, while they show dose-related side effects and uncontrolled drug release behavior. In this study, we developed biomimetic MTX-loaded ZIF-8 nanoparticles that were encapsulated inside macrophage-derived MVs, and we modified them with FPD to target them to affected joints in RA.

The successful formation of MTX@ZIF-8 was attributed to the strong interaction between the zinc ion in ZIF-8 and the carboxyl groups in MTX. After uptake by macrophages, the nanoparticles are exposed first to pH 6.0–6.5 in early endosomes and then to pH 4.5–5.5 in late endosomes and lysosomes [23]. We found that, under these weakly acidic conditions, ZIF-8 can selectively decompose and release MTX inside cells. In addition, MTX@ZIF-8 nanoparticles showed high DLE (~ 70%) and EE (~ 82%) without affecting drug activity, and their performance was much better than our previously developed drug delivery system [32, 37], suggesting that ZIF-8 is a promising nanocarrier for drug delivery. During decomposition, imidazole derivatives are released, Zinc ions are also released, and they promote osteoblast cell growth, alkaline phosphatase activity, and collagen synthesis [20], thereby stimulating bone formation and inhibiting bone resorption [23].

Given that macrophages are inflammation- and immune-associated cells that show chemotaxis and selective binding, several macrophage-mimetic nanocarriers have been explored for targeting tumors and sites of inflammation [55, 56]. Macrophage-derived MVs have been identified as a promising material for coating nanoparticles to mimic macrophages and improve active targeting and drug delivery to inflamed sites [32]. MVs can also engage in electrostatic, hydrophilic, and coordinate-covalent interactions with the ZIF-8 matrix, helping to generate stable and reliable biomimetic nanoparticles.

In this study, well-defined MV/MTX@ZIF-8 nanoparticles were prepared at a membrane-to-particle ratio of 2:1. Lower ratios would increase nanoparticle aggregation and reduce surface charge, while higher ratios would increase the thickness of the nanoparticle shell [32]. Since folate receptors are overexpressed in activated macrophages [4, 57] and have a high affinity for folic acid, we further modified the nanoparticle surface with folic acid to improve targeted drug delivery and enhance the accumulation of MTX at sites of inflammation for effective RA treatment.

The developed biomimetic nanoparticles not only protected the drug, but also mitigated phagocytic cell-dependent clearance, selectively targeted sites of joint inflammation, and facilitated targeted and intracellular drug delivery. In addition, the PEG chain in FPD and the interactions between MV and ZIF-8 stabilized the drug delivery system and prolonged time in circulation[58]. In vitro studies also showed that the MV-coated preparations released MTX in a sustained manner. FPD/MV/MTX@ZIF-8 was retained the longest in the joint tissue of CIA rats, confirming that the FPD modification increases the active targeting ability of MTX@ZIF-8 nanoparticles. FPD/MV/MTX@ZIF-8 also led to much lower hepatotoxicity than free MTX, and it significantly downregulated levels of pro-inflammatory cytokines in CIA rats. These effects were associated with significantly lower histopathological scores and reduced cartilage degeneration and articular bone destruction, indicating the potential of FPD/MV/MTX@ZIF-8 nanoparticles to be a safe, effective drug delivery system for RA treatment.

## Conclusions

We prepared an MV-encapsulated biomimetic MTX@ZIF-8 nanoplatform further modified with FPD for improved active targeting, prolonged retention, and enhanced immune evasion. The developed nanoparticles showed high DLE, pH-responsive drug release, inflammation targeting, and strong ability to inhibit inflammation as well as protect bones and cartilage in CIA rats. FPD/MV/MTX@ZIF-8 shows promise for the treatment of RA and may serve as a guide for the design of novel, more effective nanoplatforms.

## Supplementary Information

**Additional file 1: Fig. S1.** Sketch illustration of the interactions between the ZIF-8 matrix and the decorated MV. **Fig. S2.** Characterization of ZIF-8 and MTX@ZIF-8 crystals. Nitrogen adsorption isotherms of (A) ZIF-8, (B) MTX@ZIF-8. **Fig. S3.** Stability of FPD/MV/MTX@ZIF-8. The size and zeta potential change of FPD/MV/MTX@ZIF-8 over 3 days. **Fig. S4.** Ultraviolet-visible spectra of folic acid and FPD/MV/ZIF-8. **Fig. S5.** MTX@ZIF-8 nanoparticles are pH-responsive. TEM images of MTX@ZIF-8 nanoparticles incubated for (A) 1h (B) 2h in acidic buffer (pH=5.0). **Fig. S6.** Sketch illustration of the endosomal escape of FPD/MV/MTX@ZIF-8 nanoparticles by “proton sponge” effect. **Fig. S7.** The result of the in vitro cytotoxicity by (A) MTT assay and (B) CCK-8 assay at 24 h. (C) Representative images for RAW264.7 cells viability as detected by Calcein-AM/PI staining. **Fig. S8.** Uptake of Rhm B@ZIF-8. (A) Confocal microscopy showing uptake of Rhm B@ZIF-8 in RAW264.7 cells without LPS activation. (B) With LPS. **Fig. S9.** LPS-activated RAW264.7 cells were pretreated with folic acid, and cellular uptake of FPD/MV/MTX@ZIF-8 NPs was measured. **Fig. S10.** Expression of (A) tumor necrosis factor-α (TNF-α), (B) interleukin (IL)-1β, and (C) IL-10 in LPS-activated RAW264.7 cells treated with different preparations. **Fig. S11.** Hemolysis results picture of different formulations. **Fig. S12.** In vivo Cy5 fluorescence images showing the arthritic joint distribution of free Cy5, and Cy5-loaded preparations. (A) In CIA rats with inflamed joints at different time post injection. (B) Semi-quantitation of fluorescence intensity in joints. **Fig. S13.** Drug concentrations in blood. (A) Changes in blood drug concentration. (B) Pharmacokinetic parameters. **Fig. S14.** The paw thickness was recorded every 3 days. **Fig. S15.** Pro-inflammatory cytokine levels in the serum of rats with collagen-induced arthritis after treatment with different formulations. (A) Tumor necrosis factor-α (TNF-α). (B) Interleukin (IL)-1β. **Fig. S16.** Histological assessment of ankle pathology(400×). **Fig. S17.** Biochemical indexes in serum. Levels of (A) AST and (B) ALT.

## Data Availability

All data generated or analyzed during this study are included in this article.

## Abbreviations

IL: Interleukin
MTX: Methotrexate
PBS: Pphosphate-buffered saline
LPS: Lipopolysaccharide
TNF-α: Tumor necrosis factor-α
Rhm B: Rhodamine B
MOF: Metal−organic framework
MV: Microvesicle
RA: Rheumatoid arthritis
FPD: 1,2-distearoyl-*sn*-glycero-3-phosphoethanolamine-*N*-[folate(polyethylene glycol)-2000]
