# Supplementary material for: Microvesicle-camouflaged biomimetic nanoparticles encapsulating a metal-organic framework for targeted rheumatoid arthritis therapy
Source: J Nanobiotechnology. 2022 Jun 3;20:253. doi: 10.1186/s12951-022-01447-0 (PMC9164508; doi:10.1186/s12951-022-01447-0)
Supplement: Supplementary file 1 — Additional file 1: Fig. S1. Sketch illustration of the interactions between the ZIF-8 matrix and the decorated MV. Fig. S2. Characterization of ZIF-8 and MTX@ZIF-8 crystals. Nitrogen adsorption isotherms of (A) ZIF-8, (B) MTX@ZIF-8. Fig. S3. Stability of FPD/MV/MTX@ZIF-8. The size and zeta potential change of FPD/MV/MTX@ZIF-8 over 3 days. Fig. S4. Ultraviolet-visible spectra of folic acid and FPD/MV/ZIF-8. Fig. S5. MTX@ZIF-8 nanoparticles are pH-responsive. TEM images of MTX@ZIF-8 nanoparticles incubated for (A) 1h (B) 2h in acidic buffer (pH=5.0). Fig. S6. Sketch illustration of the endosomal escape of FPD/MV/MTX@ZIF-8 nanoparticles by “proton sponge” effect. Fig. S7. The result of the in vitro cytotoxicity by (A) MTT assay and (B) CCK-8 assay at 24 h. (C) Representative images for RAW264.7 cells viability as detected by Calcein-AM/PI staining. Fig. S8. Uptake of Rhm B@ZIF-8. (A) Confocal microscopy showing uptake of Rhm B@ZIF-8 in RAW264.7 cells without LPS activation. (B) With LPS. Fig. S9. LPS-activated RAW264.7 cells were pretreated with folic acid, and cellular uptake of FPD/MV/MTX@ZIF-8 NPs was measured. Fig. S10. Expression of (A) tumor necrosis factor-α (TNF-α), (B) interleukin (IL)-1β, and (C) IL-10 in LPS-activated RAW264.7 cells treated with different preparations. Fig. S11. Hemolysis results picture of different formulations. Fig. S12. In vivo Cy5 fluorescence images showing the arthritic joint distribution of free Cy5, and Cy5-loaded preparations. (A) In CIA rats with inflamed joints at different time post injection. (B) Semi-quantitation of fluorescence intensity in joints. Fig. S13. Drug concentrations in blood. (A) Changes in blood drug concentration. (B) Pharmacokinetic parameters. Fig. S14. The paw thickness was recorded every 3 days. Fig. S15. Pro-inflammatory cytokine levels in the serum of rats with collagen-induced arthritis after treatment with different formulations. (A) Tumor necrosis factor-α (TNF-α). (B) Interleukin (IL)-1β. Fig. S16. His [file 12951_2022_1447_MOESM1_ESM.doc]

# Supplementary material

**Microvesicle-camouflaged biomimetic nanoparticles encapsulating a metal-organic framework for targeted rheumatoid arthritis therapy**

*Yao Wang ^1#^, Ming Jia ^1#^, Xiu Zheng ^1#^,* *Chenglong Wang ^1^, Yun Zhou ^2^, Hong Pan ^3^, Yan Liu ^1^, Ji Lu ^4*^, Zhiqiang Mei ^5*^, Chunhong Li ^1*^*

^1^ Department of Pharmaceutical Sciences, School of Pharmacy, Southwest Medical University, Luzhou, Sichuan, China.

^2^ School of Medical Information and Engineering, Southwest Medical University, Luzhou, Sichuan, China.

^3^ Center for Medical Information and Modern Educational Technology, Southwest Medical University, Luzhou, Sichuan, China.

^4^ Department of Medicinal Chemistry, School of Pharmacy, Southwest Medical University, Luzhou, Sichuan, China.

^5^ The Research Center for Preclinical Medicine, Southwest Medical University, Luzhou, Sichuan, China.

Corresponding Author

**Ji Lu** – *Department of Medicinal Chemistry, School of Pharmacy, Southwest Medical University, 1-1 Xianglin Road, Luzhou, Sichuan, 646000, People’s Republic of China.*

*Tel: +86 19982592119.*

E-mail: [ljlyt8631@163.com](mailto:ljlyt8631@163.com)

**Zhiqiang Mei**- *The Research Center for Preclinical Medicine, Southwest Medical University, 1-1 Xianglin Road, Luzhou, Sichuan, 646000, People’s Republic of China.*

[*Tel:13795749367*](tel:13795749367)*.*

E-mail: xuguangyin1@163.com

**Chunhong Li** – *Department of Pharmaceutical Sciences, School of Pharmacy, Southwest Medical University, 1-1 Xianglin Road, Luzhou, Sichuan, 646000, People’s Republic of China.*

*Tel: +86 13679696586.*

E-mail: [lispringhong@126.com](mailto:lispringhong@126.com)


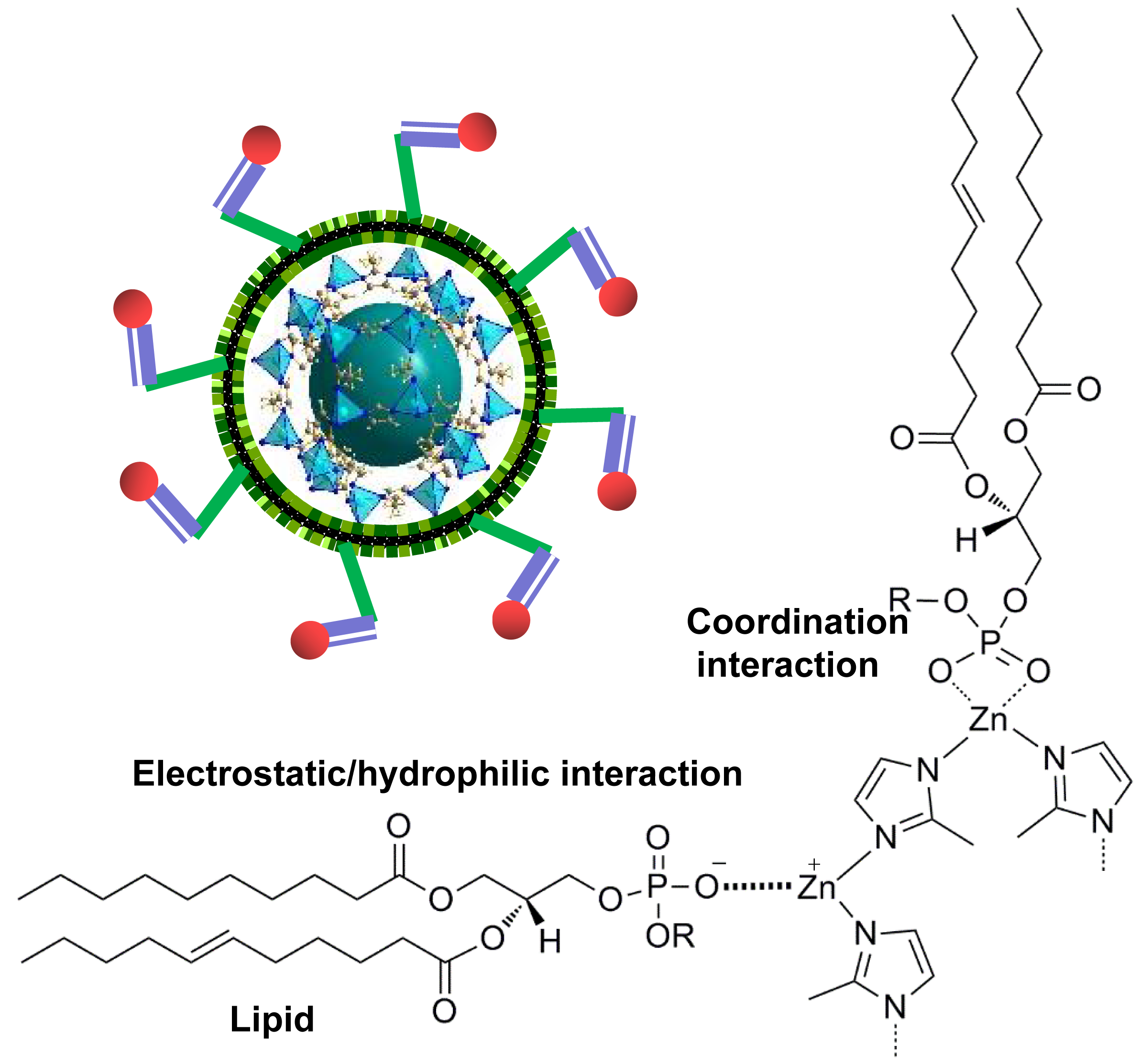


**Fig. S1.** Sketch illustration of the interactions between the ZIF-8 matrix and the decorated MV. Self-assembly of the nanoparticles could be driven by electrostatic and hydrophilic interactions between the negatively charged MV and the positively charged ZIF-8 matrix surface. Moreover, the unsaturated zinc ions on the surface of ZIF-8 matrix could also serve as the anchorages to strongly coordinate with the P-O bonds in phospholipids molecules from the MV.


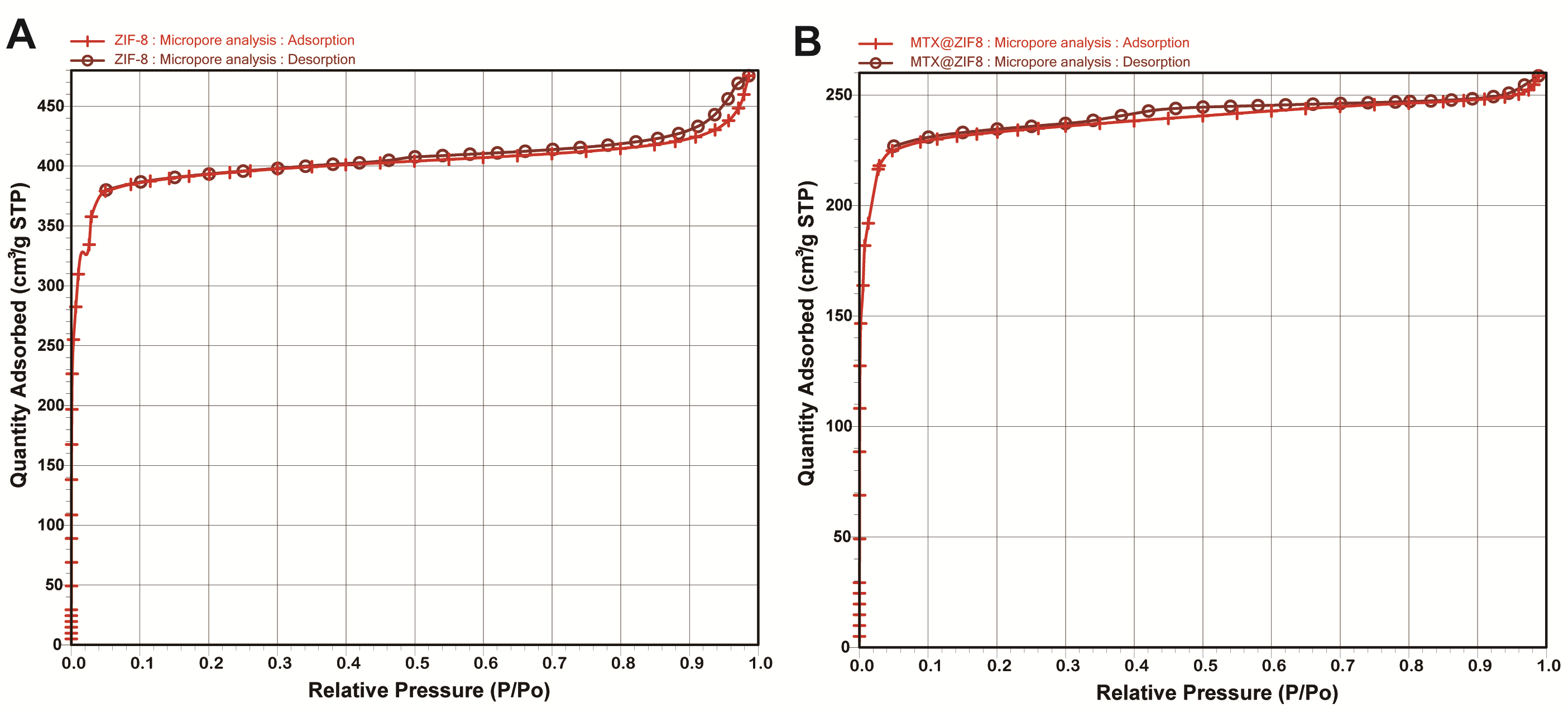


**Fig. S2.** Characterization of ZIF-8 and MTX@ZIF-8 crystals. Nitrogen adsorption isotherms of (**A**) ZIF-8, (**B**) MTX@ZIF-8.


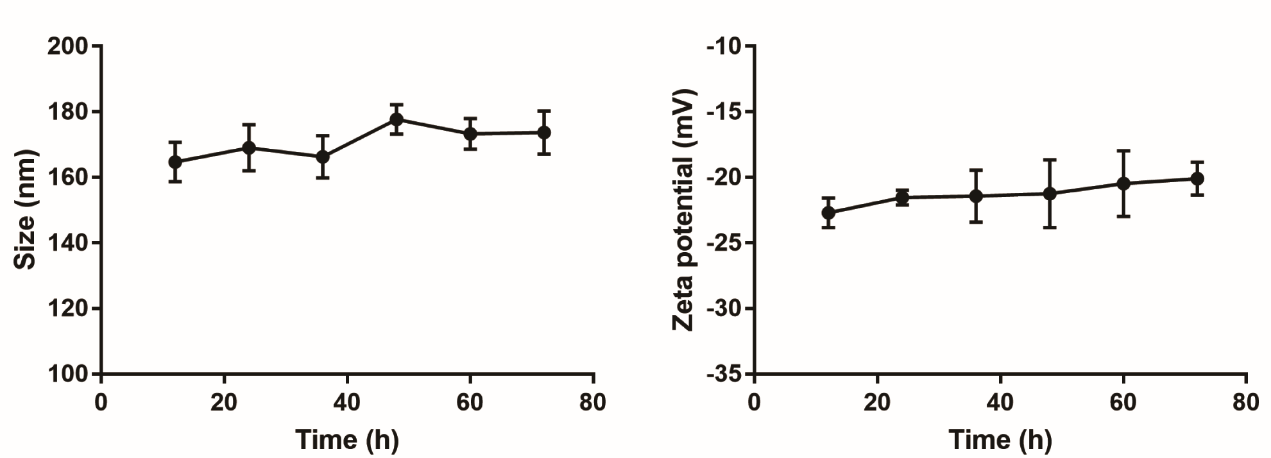


**Fig. S3.** Stability of FPD/MV/MTX@ZIF-8. The size and zeta potential change of FPD/MV/MTX@ZIF-8 over 3 days; n = 3.


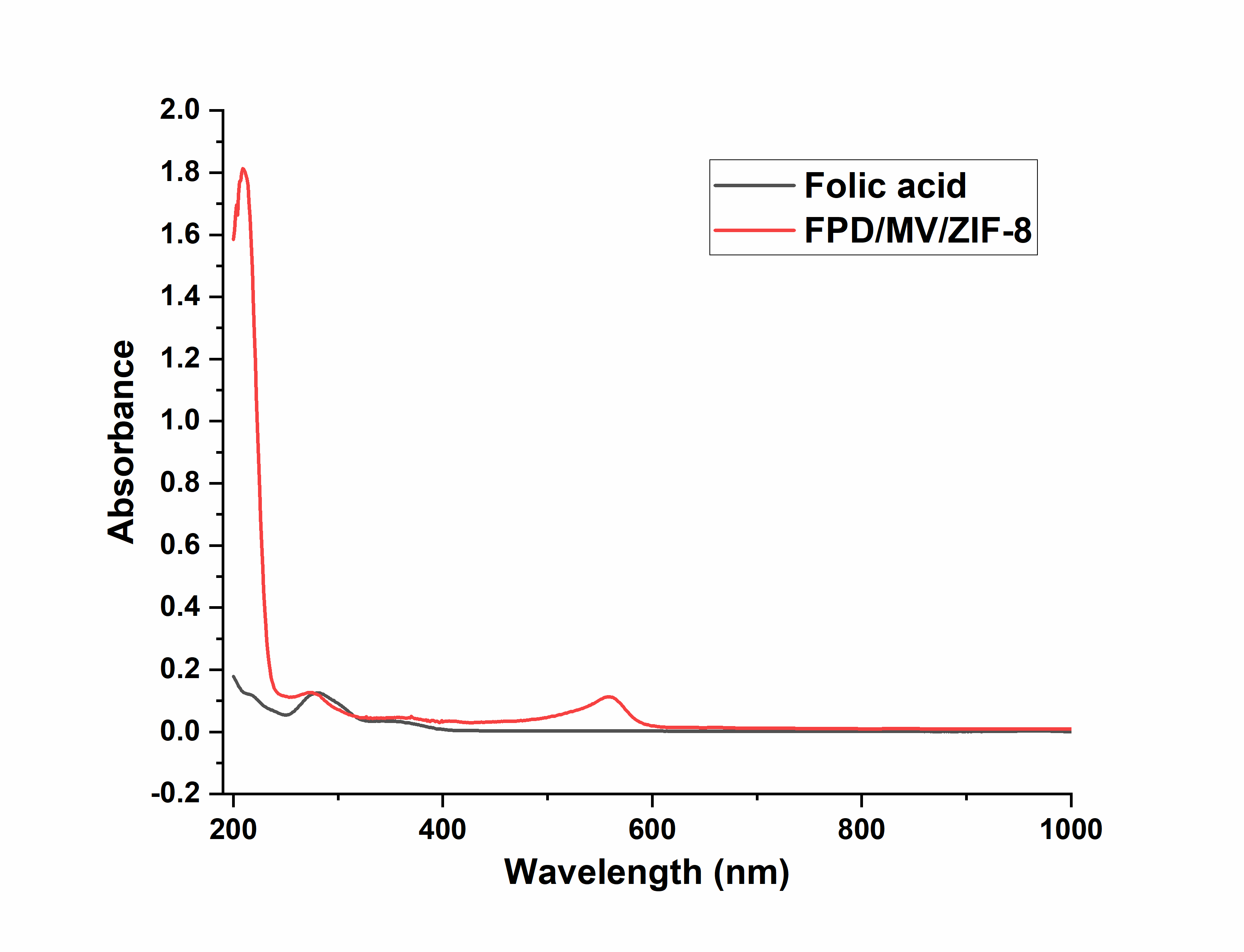


**Fig. S4.** Ultraviolet-visible spectra of folic acid and FPD/MV/ZIF-8.


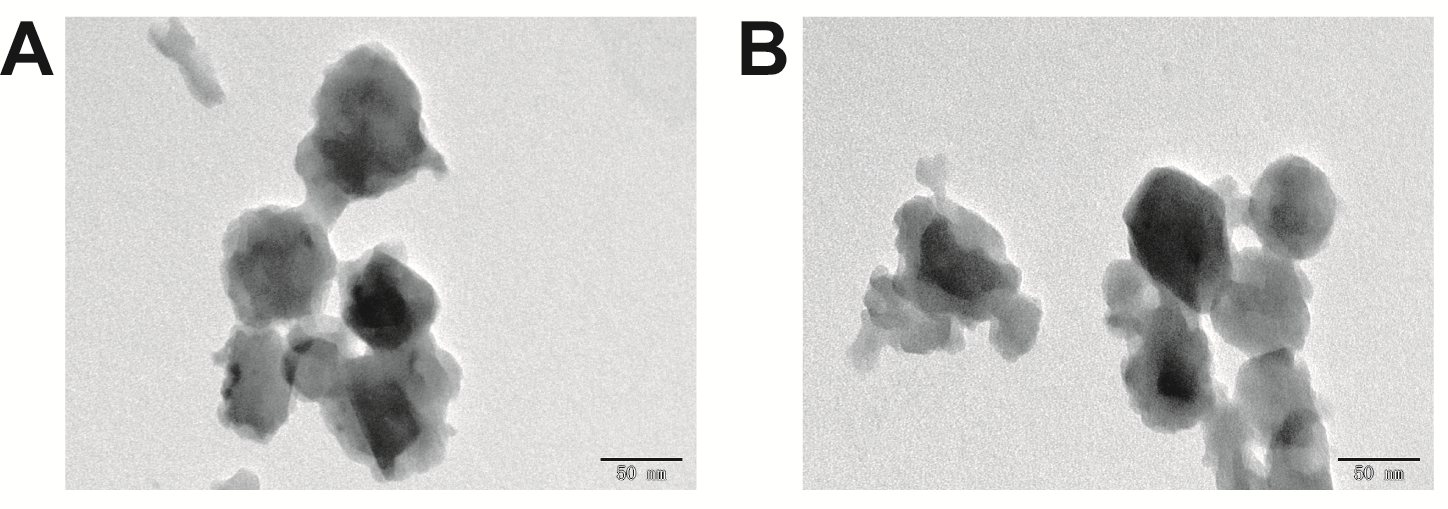


**Fig. S5.** MTX@ZIF-8 nanoparticles are pH-responsive. TEM images of MTX@ZIF-8 nanoparticles incubated for (**A**) 1h (**B**) 2h in acidic buffer (pH=5.0).


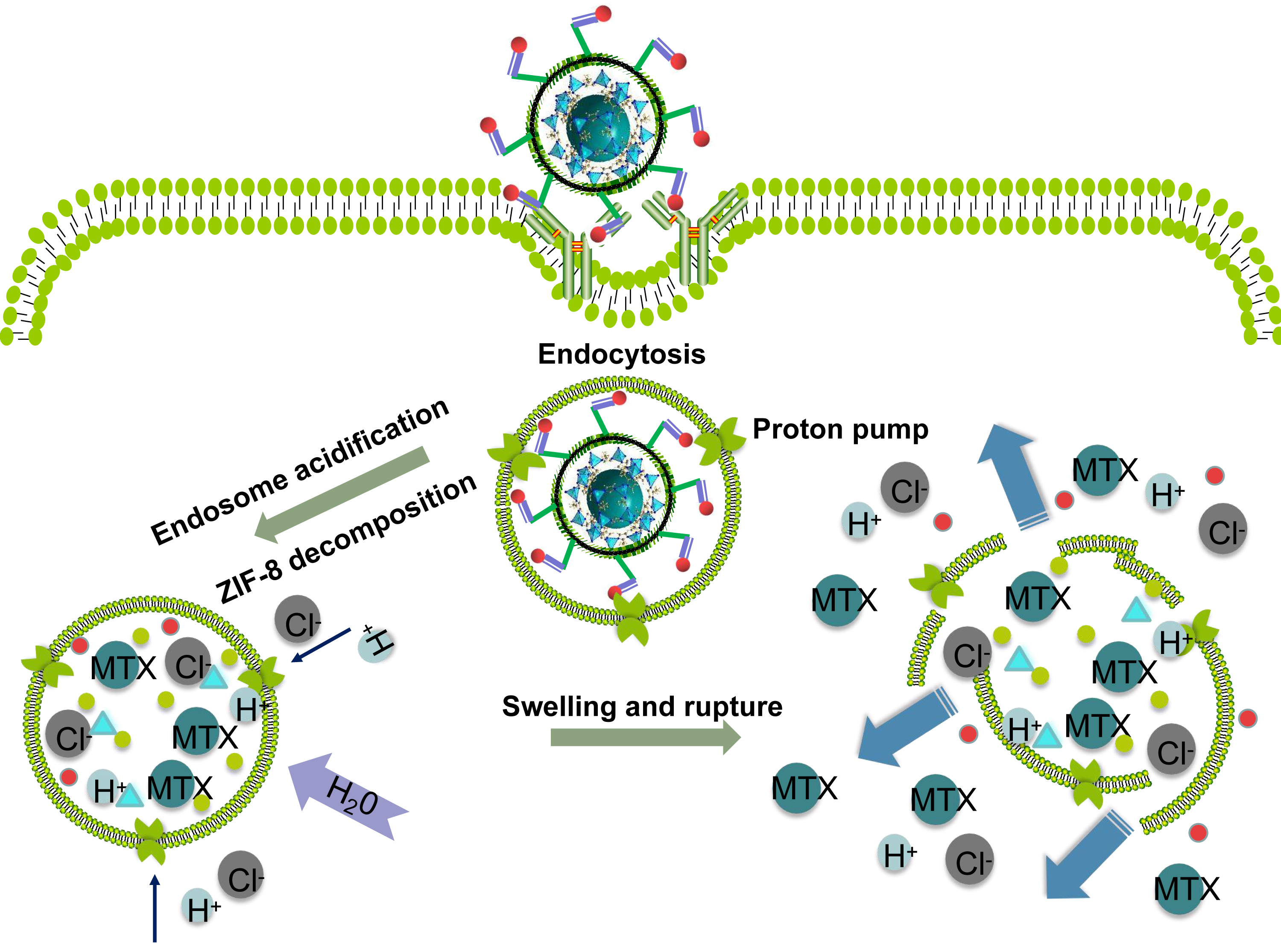


**Fig. S6.** Sketch illustration of the endosomal escape of FPD/MV/MTX@ZIF-8 nanoparticles by “proton sponge” effect. After adsorption, the designed pH-responsive nanoparticles can release organic ligands of imidazole derivatives, which can buffer the protonation of the imidazole ring. Protons accumulate along with their counterions in the endoplasm, stimulating the entry of water from the cytoplasm to balance the high osmotic pressure in the endoplasm. In the presence of ligands, the expansion of the content body eventually leads to the rupture of the content body and the release of the guest molecules.


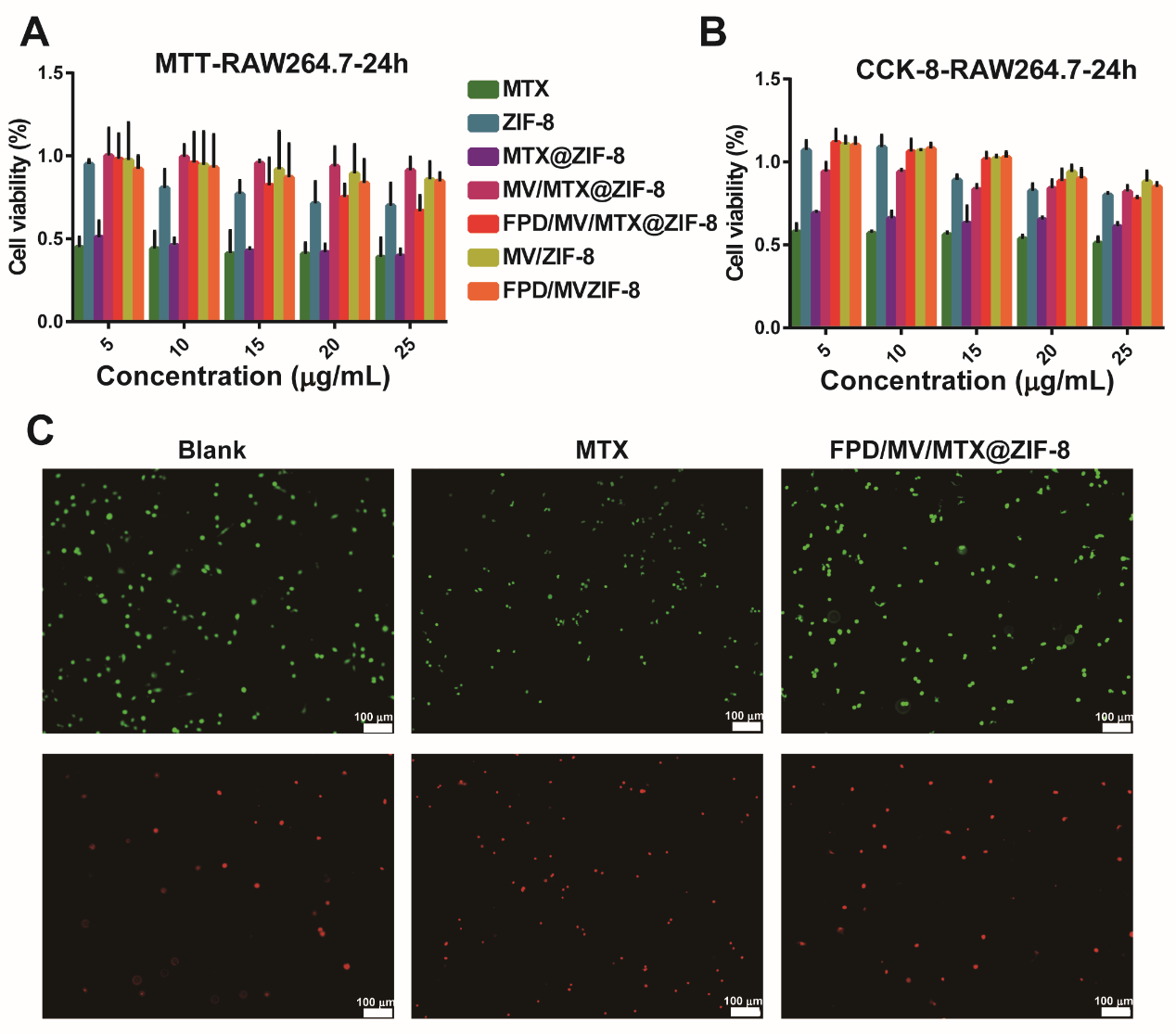


**Fig. S7.** The result of the in vitro cytotoxicity by (A) MTT assay and (B) CCK-8 assay at 24 h. (C) Representative images for RAW264.7 cells viability as detected by Calcein-AM/PI staining. Green for Calcein-AM fluorescence represents viable cells, red for PIfluorescence represents dead cells.


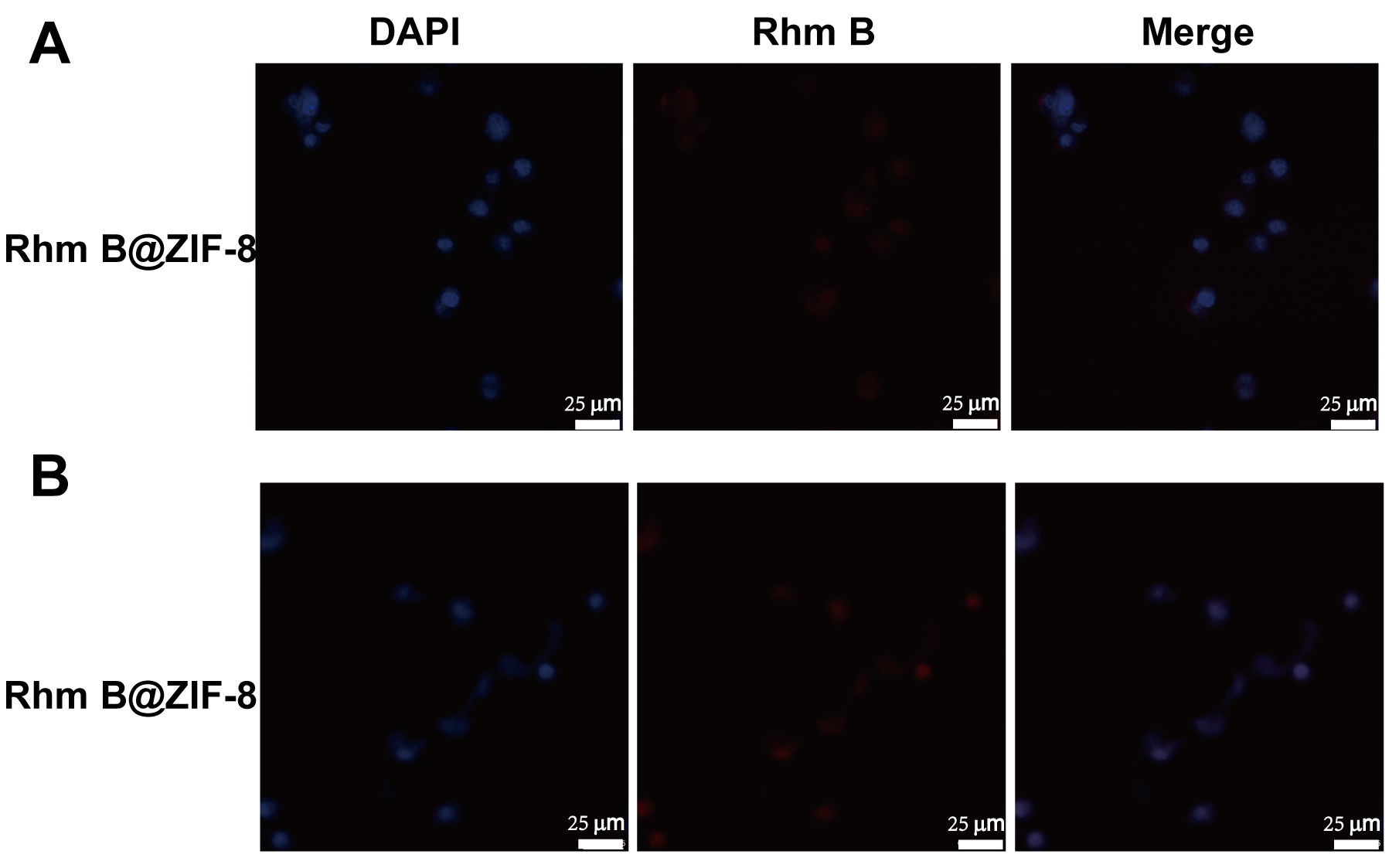


**Fig. S8.** Uptake of Rhm B@ZIF-8. (**A**) Confocal microscopy showing uptake of Rhm B@ZIF-8 in RAW264.7 cells without LPS activation. (**B**) With LPS. Scale bar: 25 μm.


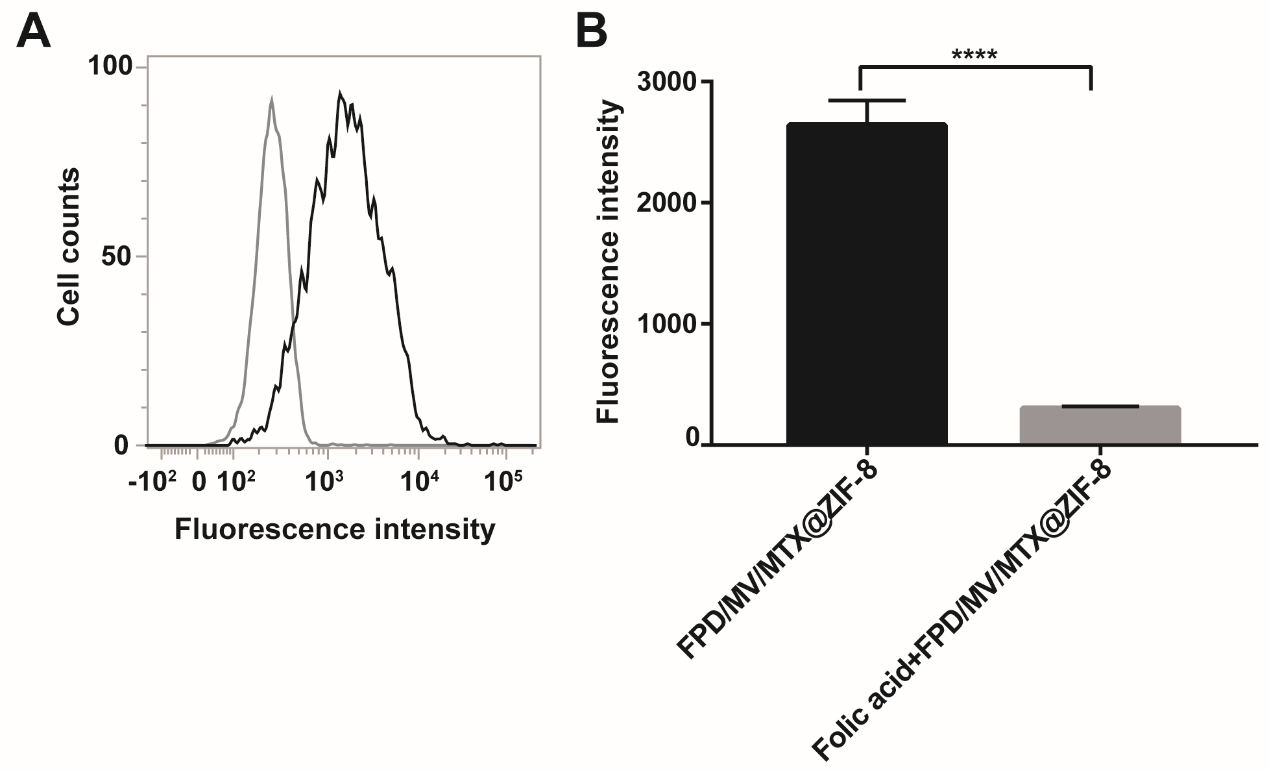


**Fig. S9.** LPS-activated RAW264.7 cells were pretreated with folic acid, and cellular uptake of FPD/MV/MTX@ZIF-8 NPs was measured.


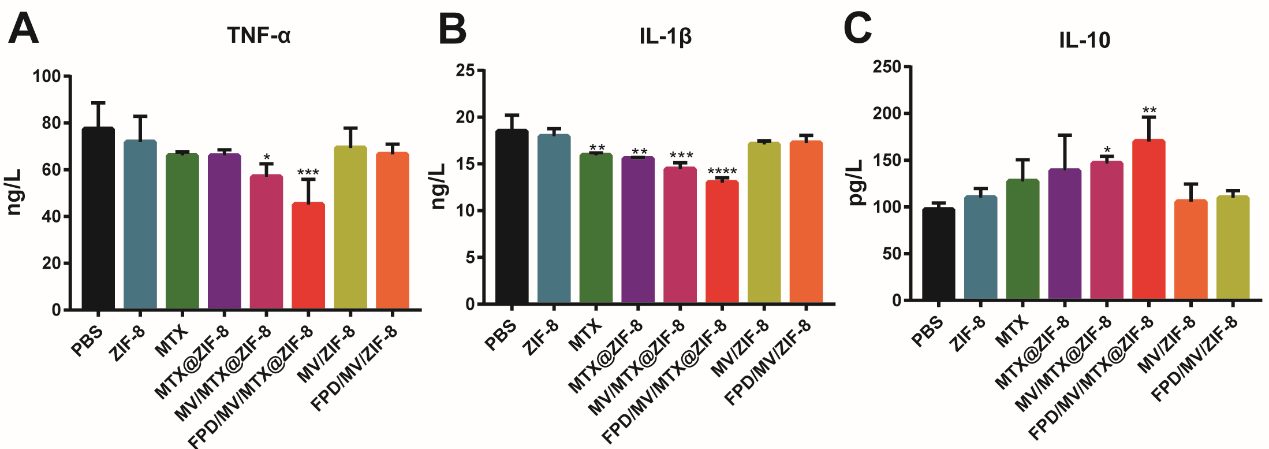


**Fig. S10.** Expression of (**A**) tumor necrosis factor-α (TNF-α), (**B**) interleukin (IL)-1β, and (**C**) IL-10 in LPS-activated RAW264.7 cells treated with different preparations. Data are shown as mean ± SD (n = 3). ^*^*P* < 0.05, ^**^*P* < 0.01, ^***^*P* < 0.001, ^****^*P* < 0.0001. FPD, 1,2-distearoyl-*sn*-glycero-3-phosphoethanolamine-*N*-[folate (polyethylene glycol)-2000; MTX, methotrexate; MV, microvesicle; PBS, phosphate-buffered saline; ZIF-8, zeolitic imidazolate framework-8.


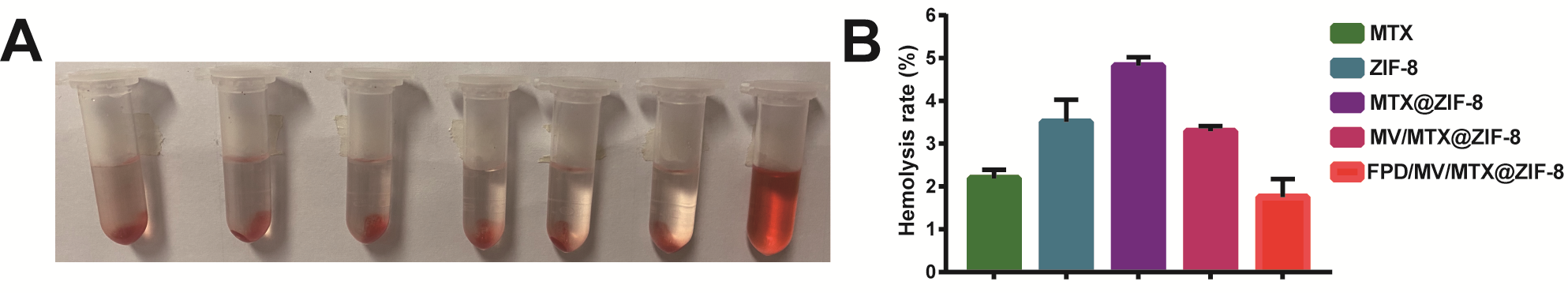


**Fig. S11.** Hemolysis results picture of different formulations. (**A**) From left to right are normal saline, MTX, ZIF-8, MTX@ZIF-8, MV/MTX@ZIF-8, FPD/MV/MTX@ZIF-8 and UP water. (**B**) Hemolysis rate analysis of different formulations. Results were shown as mean ± SD (n = 3).


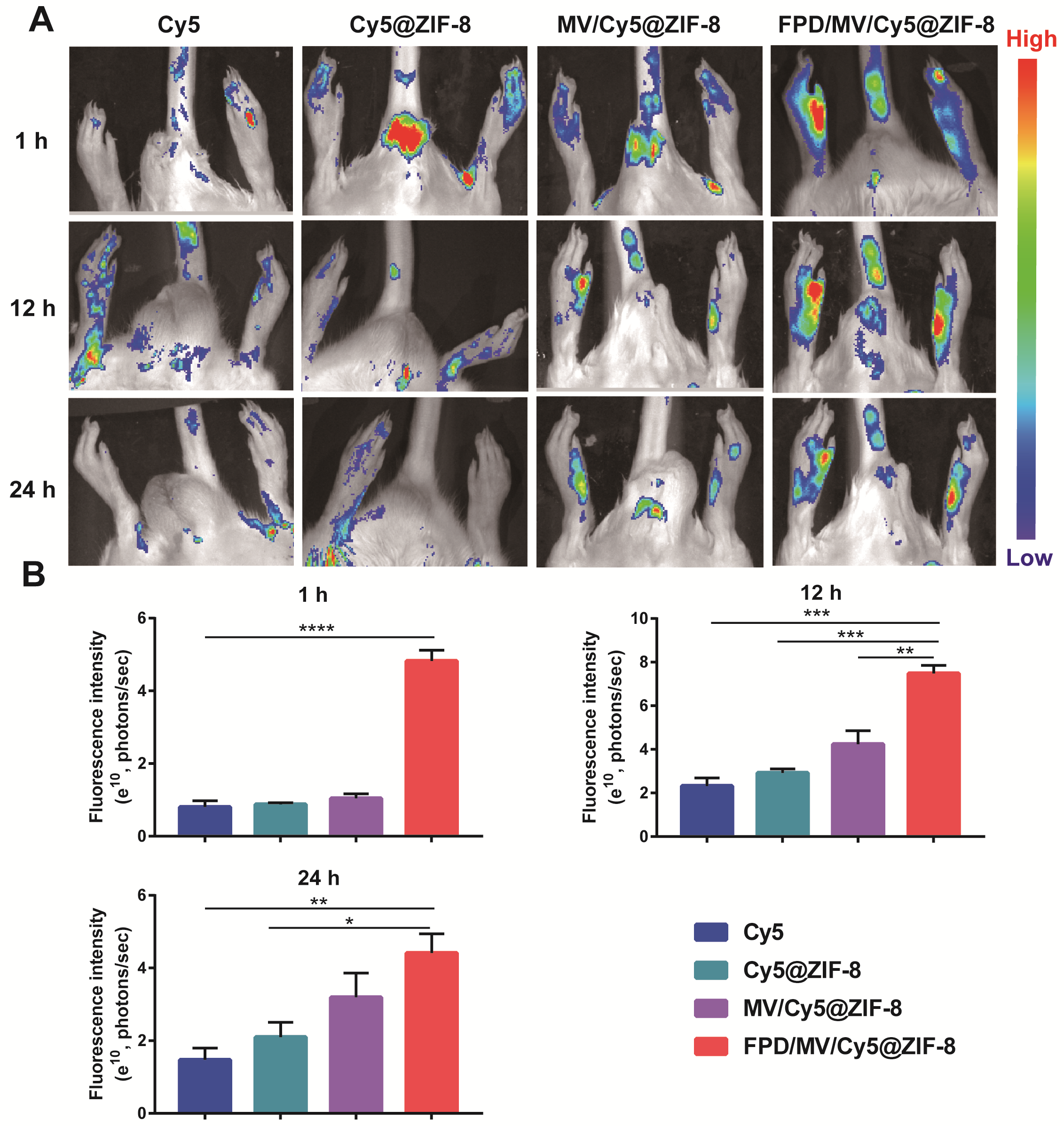


**Fig. S12.** In vivo Cy5 fluorescence images showing the arthritic joint distribution of free Cy5, and Cy5-loaded preparations. (**A**) In CIA rats with inflamed joints at different time post injection. (**B**) Semi-quantitation of fluorescence intensity in joints. Results were shown as mean ± SD (n = 3). *P < 0.05, **P < 0.01, ***P < 0.001, ****P < 0.0001.


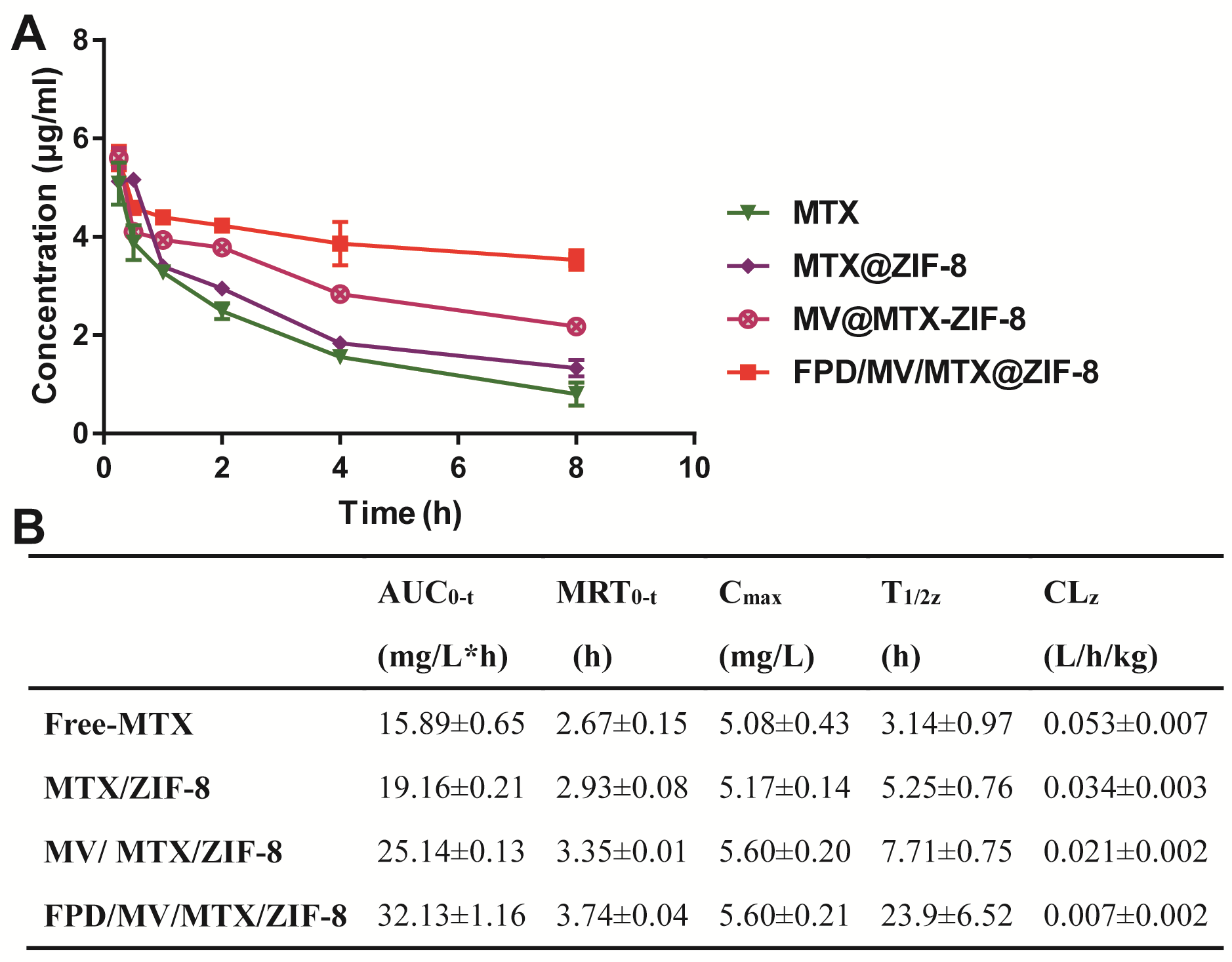


**Fig. S13.** Drug concentrations in blood. (**A**) Changes in blood drug concentration. (**B**) Pharmacokinetic parameters. Results were shown as mean ± SD (n = 3).


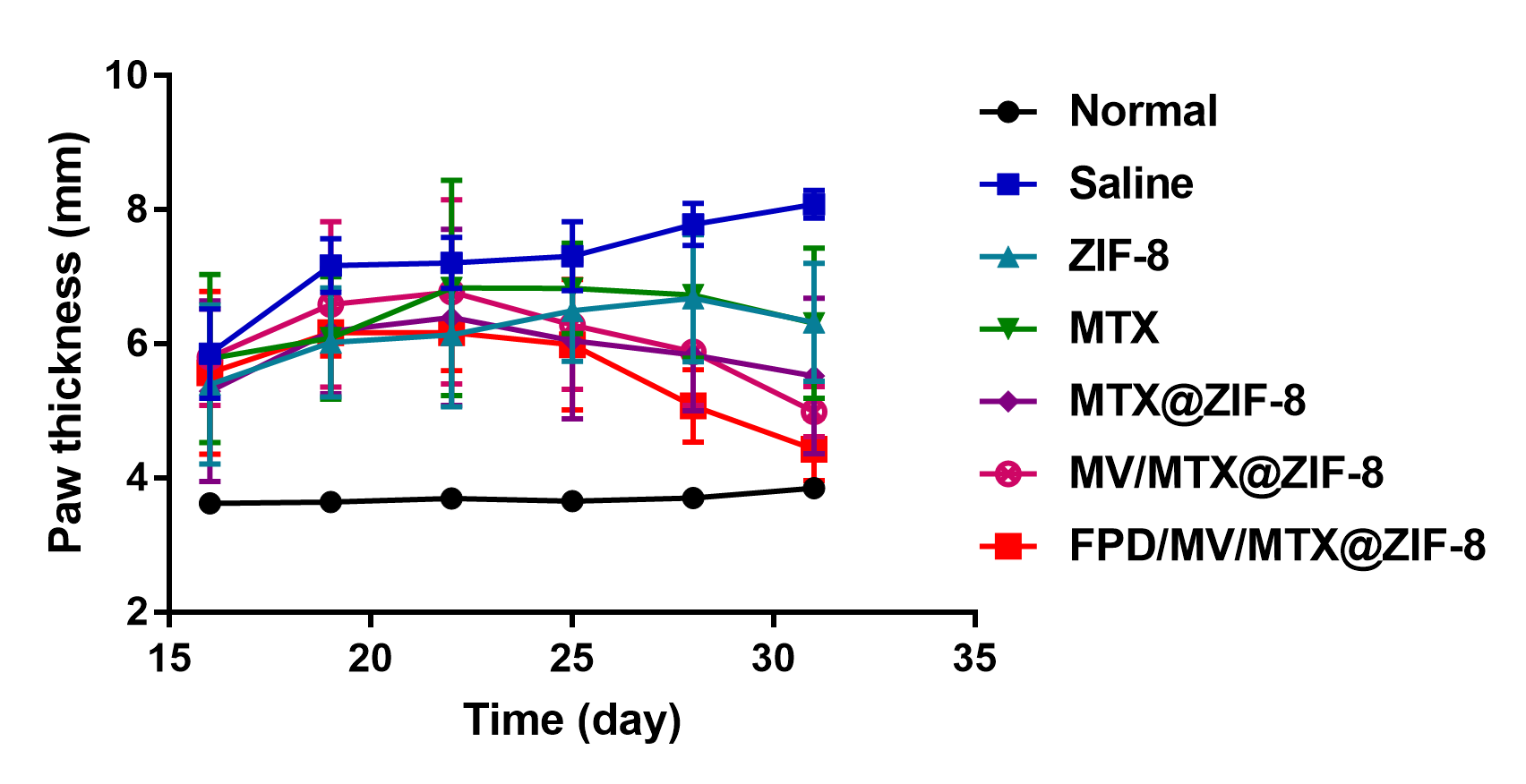


**Fig. S14.** The paw thickness was recorded every 3 days. Results were shown as mean ± SD (n = 3).


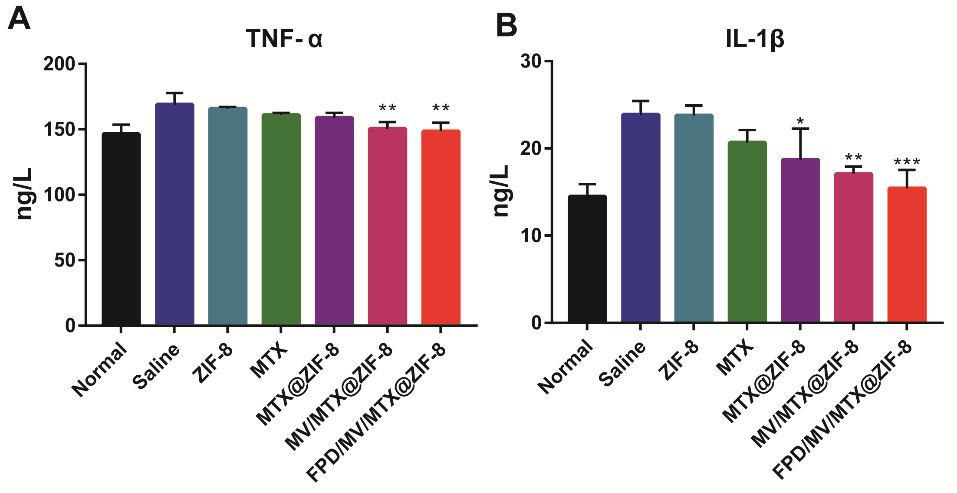


**Fig. S15.** Pro-inflammatory cytokine levels in the serum of rats with collagen-induced arthritis after treatment with different formulations. (**A**) Tumor necrosis factor-α (TNF-α). (**B**) Interleukin (IL)-1β. Data are shown as mean ± SD (n = 3). ^*^*P* < 0.05, ^**^*P* < 0.01, ^***^*P* < 0.001 *vs* saline group. FPD, 1,2-distearoyl-*sn*-glycero-3-phosphoethanolamine-*N*-[folate (polyethylene glycol)-2000; MTX, methotrexate; MV, microvesicle; ZIF-8, zeolitic imidazolate framework-8.


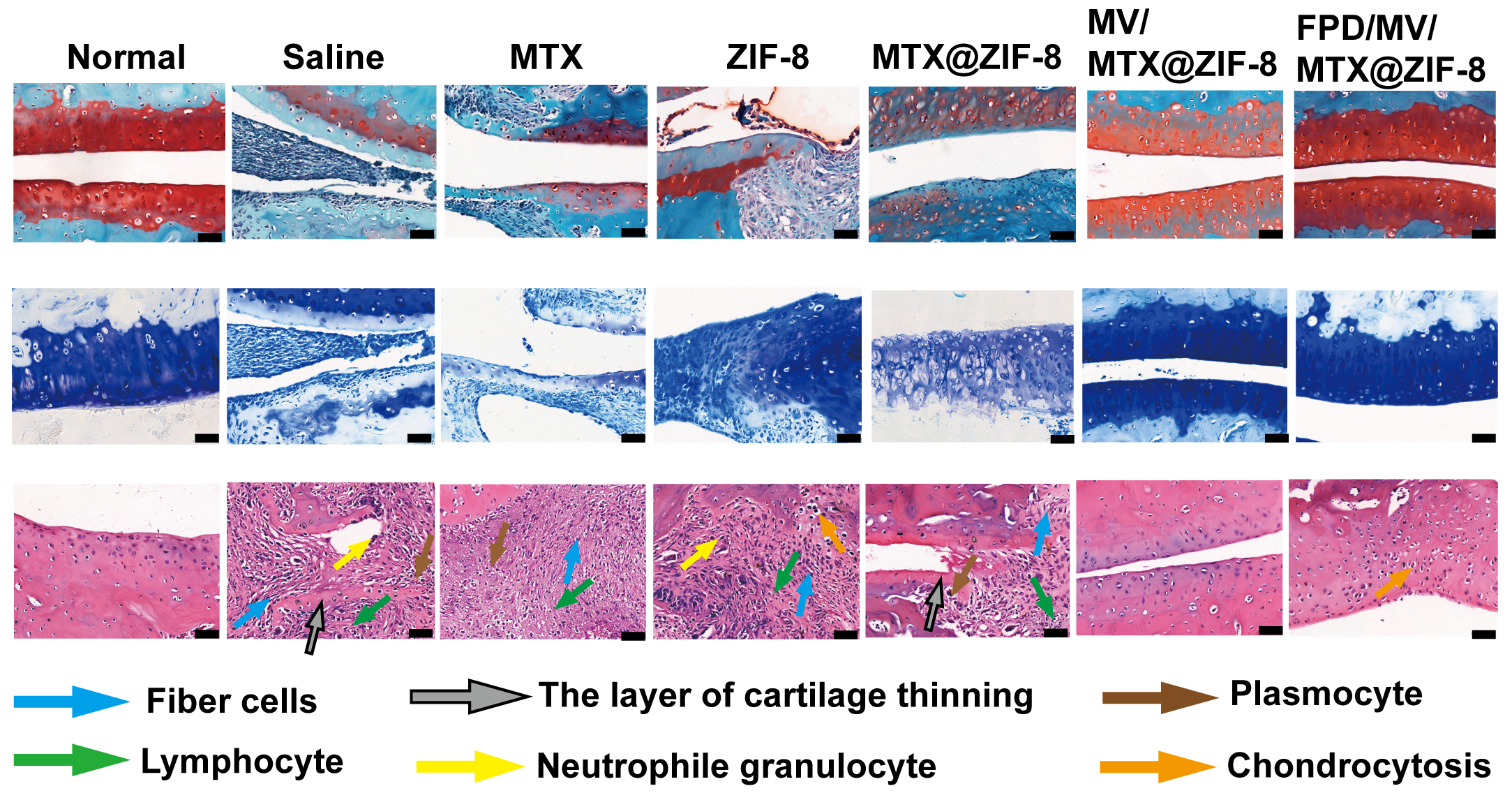


**Fig. S16.** Histological assessment of ankle pathology**.** Representative photographs of hind limbs at the endpoint of the experiment from different treatment groups; histopathology evaluation of ankle joints were identified using safranin O, toluidine blue staining and H&E (400×). Scale bar: 50 μm.


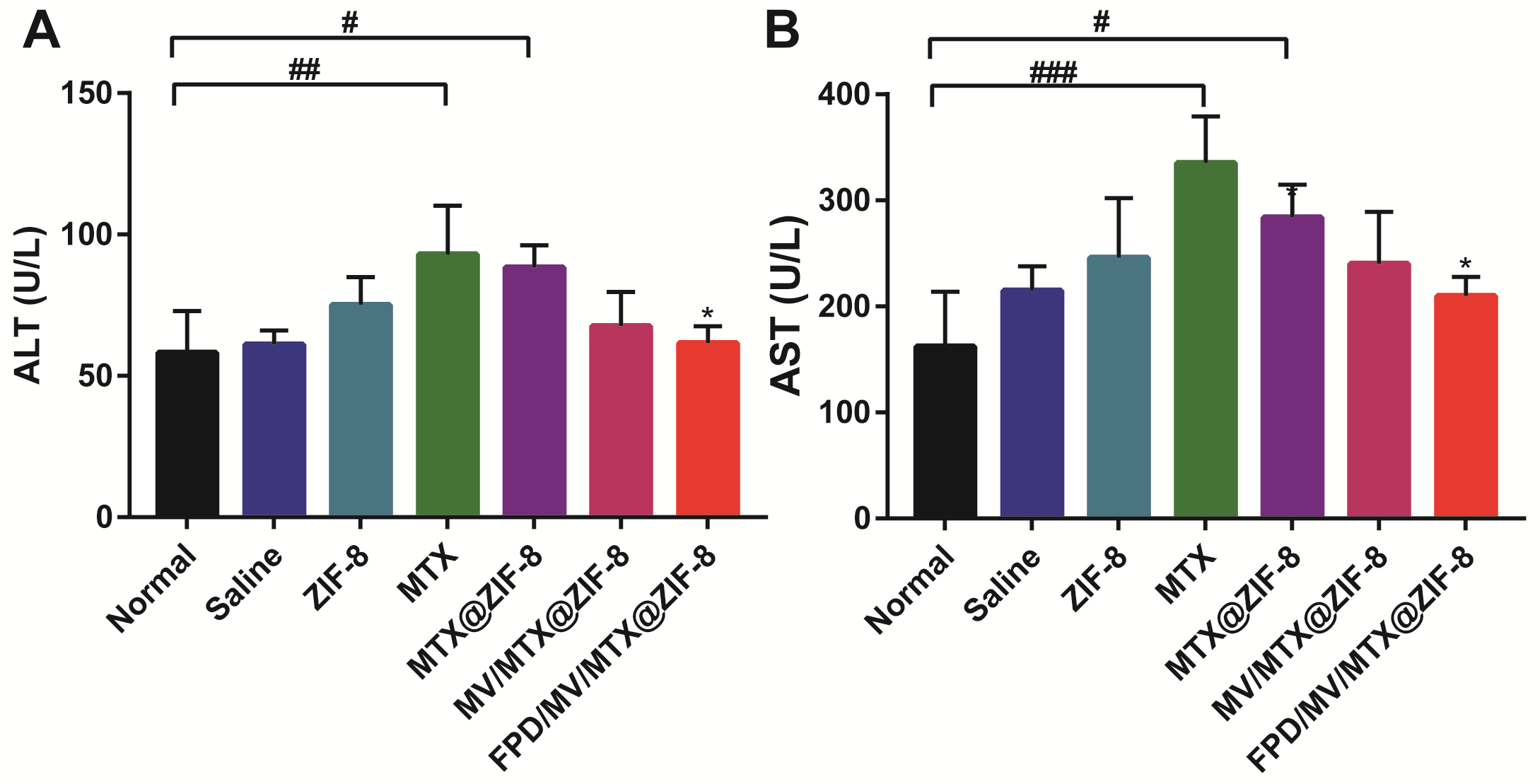


**Fig. S17.** Biochemical indexes in serum. Levels of (**A**) AST and (**B**) ALT. Results were shown as mean ± SD (n = 3). ^#^P < 0.05, ^##^P < 0.01, ^###^P < 0.001, ^*^P < 0.05 vs rats treated with MTX.
